# The Ethics of Leveraging Routinely Collected Patient Data for AI Development: Mixed Methods Study

**DOI:** 10.2196/79863

**Published:** 2026-03-02

**Authors:** Menno T Maris, Joanna E Klopotowska, Ronald Cornet, Mariëtte A Van den Hoven, Joris E Lieverse, Daniel Fernández-Llaneza, Marieke A R Bak, LEAPfROG Consortium, Ameen Abu-Hanna

**Affiliations:** 1 Department of Ethics, Law and Humanities, Amsterdam UMC Location University of Amsterdam, Meibergdreef 9, Amsterdam, The Netherlands, 31 (020) 566 9111; 2 Amsterdam Public Health Institute, Amsterdam, The Netherlands; 3 Department of Medical Informatics, Amsterdam UMC location University of Amsterdam, Amsterdam, The Netherlands; 4 Department of Ethics, Law and Humanities, Amsterdam UMC location Vrije Universiteit Amsterdam, Amsterdam, The Netherlands; 5 Institute of History and Ethics in Medicine, TUM School of Medicine, Technical University of Munich, Munich, Germany; 6 See Acknowledgments

**Keywords:** artificial intelligence, routinely collected health data, ethics, stakeholder participation, medical informatics, pharmacotherapy

## Abstract

**Background:**

Electronic health record (EHR) data, a key form of routinely collected patient data, offer great potential for medical research and the development of artificial intelligence (AI) tools. However, because these data are primarily gathered for health care rather than research, it often lacks the quality needed for AI training, raising both methodological and ethical concerns. While previous studies have reviewed the ethical implications of both routinely collected patient data and AI separately, their intersection, where AI is applied to such data, remains largely unexplored.

**Objective:**

This study aimed to examine the ethical challenges that arise at the intersection of EHR data and AI development and to derive practice-oriented recommendations using the Dutch LEAPfROG (Leveraging Real-World Data to Optimize Pharmacotherapy Outcomes in Multimorbid Patients Using Machine Learning and Knowledge Representation Methods) project as a guiding case.

**Methods:**

We used a mixed methods design combining a scoping literature review with a systematic search and 2 stakeholder workshops structured by the guidance ethics approach, reflecting a staged and iterative process aligned with the LEAPfROG project’s development phases. The review identified 25 relevant publications from 2014 to 2024. The workshops, conducted with 17 and 13 participants respectively, included patients, clinicians, ethicists, data officers, and AI developers. Both workshops used dialogue to identify ethical values, impacts, and action points, focusing on a case study of drug-induced acute kidney injury.

**Results:**

The analysis highlighted four major themes: (1) data privacy, transparency, and consent, including challenges of meaningful consent and risks of reidentification; (2) public trust and regulatory challenges, such as fragmented oversight and inconsistent governance; (3) fair representation and model generalizability, where incomplete or biased data may perpetuate health inequities; and (4) responsible AI integration in clinical practice, including concerns about clinical tropism, administrative burden, and the risk of overreliance on AI outputs. Both literature and stakeholder perspectives underscore the risk of decontextualization when EHR data are reused and emphasize the importance of clearly defining the purpose of data reuse to ensure real-world applicability and foster trust.

**Conclusions:**

Responsible AI development requires explicit attention to how EHR data are produced, interpreted, and governed in practice, recognizing that data quality and meaning are shaped by the clinical, institutional, and social contexts in which they originate. Technical solutions or top-down regulation alone are insufficient. Instead, stakeholder-led and context-sensitive approaches are needed to define the purposes, risks, and benefits of medical AI. Grounded in the realities of health care practice and in the perspectives of patients, clinicians, and data custodians, these approaches can strengthen transparency, fairness, and clinical relevance, ensuring that EHR data are used ethically and effectively to support equitable and trustworthy AI innovation.

## Introduction

Both routinely collected patient data and artificial intelligence (AI) are widely regarded as promising resources for advancing medical research. AI methods are valued for their ability to manage and analyze large, diverse datasets, making them potentially well-suited to maximize the potential of routinely collected patient data. Studies leveraging such data offer several advantages over other study designs, including lower administrative costs, adaptability to evolving practice patterns, and access to larger sample sizes [1]. One of the fields where leveraging routinely collected data with AI seems particularly promising is individualized pharmacotherapy, focusing on the “rational use of drugs” to tailor pharmacotherapy to patients’ clinical needs, thereby maximizing benefits and minimizing harm [2]. Effective innovation in pharmacotherapy is currently especially challenging for patients with multimorbidity, characterized by the presence of 2 or more chronic diseases and a frequent need for multiple medications, a phenomenon known as polypharmacy [3,4]. Despite the increasing prevalence of multimorbidity, partly due to population aging and improvements in health care services [5], clinical programs, guidelines, and research continue to primarily focus on managing individual diseases [3,6].

By leveraging routinely collected patient data from electronic health records (EHRs) with AI, the Dutch LEAPfROG Project (Leveraging Real-World Data to Optimize Pharmacotherapy Outcomes in Multimorbid Patients Using Machine Learning and Knowledge Representation Methods) seeks to enhance medication safety for patients with multimorbidity. A central focus of the project is the clinically urgent use case of drug-induced acute kidney injury (DAKI) in patients with chronic kidney disease (CKD). Ultimately, LEAPfROG aims to develop high-quality and fit-for-purpose EHR data, adhering to the principles of findability, accessibility, interoperability, and reusability [7] and to develop robust analytic methods, providing real-world evidence on pharmacotherapy outcomes and contributing to learning health systems that offer personalized treatments, improve quality of life, and reduce health care costs (Textbox 1).

Textbox 1. **LEAPfROG (Leveraging Real-World Data to Optimize Pharmacotherapy Outcomes in Multimorbid Patients Using Machine Learning and Knowledge Representation Methods) project: improving drug safety in chronic kidney disease (CKD) through electronic health record (EHR) data and artificial intelligence (AI).**

**Background**

The LEAPfROG project leverages routinely collected EHR data and structured domain knowledge to improve medication safety for patients with multimorbidity, specifically aiming to reduce the risk of drug-induced acute kidney injury (DAKI) in patients with CKD.

The LEAPfROG approach combines data from 3 EHR sources: detailed EHRs from Amsterdam UMC hospitals, EHRs from the Academic Network of General Practices Amsterdam (ANHA), and EHRs from general practices and outpatient pharmacies within the PHARMQ Database Network, covering general practices, outpatient pharmacies, and hospitals. Together, these datasets provide a rich source of insights into patient pharmacotherapy outcomes and drug exposures. To enhance these insights, LEAPfROG integrates domain knowledge from resources such as drug monographs, medication safety alerts, and scientific literature.

A key goal of the project is to develop transparent, explainable tools that can assist clinicians in making well-informed prescribing decisions. LEAPfROG's AI models combine machine learning with knowledge representation methods to detect DAKI, assess DAKI risk in patients with CKD, and clarify the underlying factors contributing to kidney safety issues. By doing so, the project aims to improve the detection of adverse drug events such as DAKI and enhance patient outcomes, particularly for patients with multimorbidity, such as those with CKD.

**Causal machine learning model for retrospective diagnosis of DAKI**

One of LEAPfROG's aims is the development of a causal machine learning model to assist physicians in determining whether acute kidney injury was caused by a drug (combination), a process known as “retrospective diagnosis.” This model aims not only to identify CKD patients who developed DAKI but also to explain cause-and-effect relationships underlying the DAKI diagnosis. Through this effort, LEAPfROG aims to create a large dataset of patients who did and did not develop DAKI. This dataset is critical for training future machine learning models capable of identifying patients at risk of DAKI ("prognostic models"), ultimately helping to prevent such adverse drug events in future CKD patients.

Because EHR data are collected during routine care for clinical purposes, they often have quality limitations when reused for research, lacking the consistency required by clinical research standards [8,9]. Integrating AI with EHR data may therefore exacerbate or further obscure existing data shortcomings, ultimately impacting their meaningful use in clinical practice [10,11].

Previous studies have systematically reviewed ethical issues related to leveraging health data for medical research, including concerns related to the sharing and linkage of health data [12], public attitudes towards the reuse of health data for research [13], and broader ethical considerations of using EHRs for biomedical research [14]. Regarding the ethical implications of AI in health care, previous studies have reviewed both epistemic ethical issues (ie, the type of knowledge AI generates) and normative ethical concerns [15], along with empirical investigations into ethical considerations specific to medical AI [16]. However, literature reviews on the ethical considerations inherent in the integration of EHR data and AI technologies are currently nonexistent.

In this study, we aim to explore the ethical implications of reusing EHR data for AI-driven research and innovation in health care and to provide actionable recommendations based on our use case, the LEAPfROG project. This article presents the results of a mixed methods approach that combines a scoping literature review with a systematic search and 2 innovative stakeholder engagement workshops grounded in the guidance ethics approach (GEA) [17]. The scoping review informed the design of the stakeholder workshops, which in turn generated empirical insights that complemented and contextualized the review’s findings.

## Methods

### Study Design

The study followed an iterative, participatory design that combined a scoping review and stakeholder engagement workshops to explore the ethical implications of reusing EHR data for AI-driven health research. The scoping review was conducted in accordance with the PRISMA (Preferred Reporting Items for Systematic Reviews and Meta-Analyses) extension for scoping reviews [18]. No protocol for the scoping review component of this study was registered. The stakeholder workshops were structured using the GEA for evaluating emerging technologies [17]. The overall design was staged and sequential rather than triangulated, with each step reflecting a different stage of the LEAPfROG project. This iterative process reflected the real-world complexity of AI ethics, allowing themes to unfold progressively, from broad reflection on EHR reuse to more specific considerations of AI development and implementation.

The workshops were integrated into the staged structure of the project, with their timing and number determined in advance and aligned with key project phases. Rather than aiming for thematic saturation, the workshops served as structured points of ethical reflection tied to the specific technology and context [17]. The scoping review identified ethical themes in the literature, providing a broad understanding of existing ethical considerations, while the workshops enabled further exploration and contextualization within the real-world context of the LEAPfROG project.

In the first workshop, participants discussed the ethical implications of reusing EHR data in the Dutch context, focusing on DAKI in patients with CKD. As these discussions aligned with initial literature findings, the subsequent scoping review shifted focus from the ethics of EHR reuse to ethical considerations at the intersection of EHR data and AI. In the second workshop, preliminary findings from the literature were presented to validate, contextualize, and further refine the identified themes. Furthermore, this workshop centered on a concrete AI-based tool within the LEAPfROG project: a causal machine learning model for the retrospective diagnosis of DAKI in patients with CKD.

### Scoping Review

#### Search Strategy

MTM, JEK, RC, and MB jointly developed the search strategy to identify articles that thoroughly examine or discuss the ethical considerations associated with leveraging EHR data for advancing medicine using AI. A comprehensive search was conducted by MTM in PubMed, CINAHL (via EBSCO), and Web of Science, restricted to results between March 2014 and March 2024. The search was structured around 4 key concepts: “Artificial Intelligence,” “Ethics,” “Research,” and “Routinely collected patient data” (Multimedia Appendix 1). Additionally, the most relevant MeSH (Medical Subject Headings) terms, synonyms, and related terms were identified, leading to the development of final search strings for each database. Results from the 3 databases were merged in EndNote (version 21; Clarivate Analytics), and duplicates were removed.

#### Selection Process and Analysis

One researcher (MTM) screened titles and abstracts using Rayyan [19]. Eligible studies were written in English and addressed ethical considerations at the intersection of AI, including machine learning and natural language processing, that analyze routinely collected patient data. Following Benchimol et al [20], such data were defined as health data collected without specific a priori research questions, including observational data from EHRs and disease registries. Particular emphasis during selection was placed on studies using EHRs or data comparable in origin, as these form the primary data source within the LEAPfROG project and are central to the ethical questions explored in this study. Because terminology varies across publications, the search strategy was intentionally broad to capture studies referring to similar data under related terms (eg, clinical or electronic medical records). Studies that also discussed other data sources (eg, clinical trial or wearable data) were included, provided the focus was sufficiently on the reuse of EHRs. Any uncertainties during full-text assessment were resolved by consensus between 2 researchers (MTM and MB). Reference lists were screened for additional relevant studies.

We uploaded full-text articles to MaxQDA and performed open coding to explore the ethical implications of using routinely collected EHR data for AI-driven research and innovation. Data extraction and coding were treated as the data charting process for the scoping review. Using a subset of 10 articles, one researcher (MTM) developed a coding scheme based on overlapping ethical considerations and emerging themes, such as “privacy” and “doctor-patient relationship.” This framework was discussed with the second researcher (MB) throughout the process and guided the coding of the remaining articles by MTM.

Insights from the scoping review informed both the design and thematic focus of the second stakeholder workshop. The resulting data were analyzed inductively to explore how participants interpreted and prioritized ethical themes. This analysis refined and contextualized the review’s findings, emphasizing those most pertinent to real-world practice within the LEAPfROG project and completing the staged, iterative process.

### Guidance Ethics Workshops

#### Workshop Design

We organized 2 stakeholder engagement workshops using the GEA (translated from the Dutch “Aanpak Begeleidingsethiek”), a relatively new method for addressing the ethical aspects of technological innovation [17]. The workshops were conducted in Dutch. The GEA uses dialogue to map the most pressing moral considerations in technology implementation, as identified by stakeholders [21]. The GEA primarily serves to guide the development of a technology by prioritizing ethical considerations relevant to the specific case, while striving to align with stakeholder values.

Both workshops, which lasted 3 hours each, followed the GEA approach and comprised three separate stages: (1) providing a clear description of the technology and its context, (2) identifying stakeholders, potential impacts, and underlying values, and (3) generating a list of options for responsible innovation and implementation from 3 distinct perspectives: technology, environment, and user (Textbox 2). The first workshop was prepared by JEK, RC, MTM, and MB and moderated by external facilitators from Stichting ECP. The second was prepared by JEK, MTM, MB, MvdH, and DF-L and moderated by JEK, MTM, MvdH, JEL, and MB.

Textbox 2. **Stakeholder workshops design “guidance ethics approach.”**

Stage 1: Technology in context

- Introduction by project leaders: Description and use context.
- Stakeholder inquiry: Opportunity for participants to gain a deeper understanding of and explore the details of the intended technology.

Stage 2: Dialogue

- Actors: Individuals or groups involved in or affected by the proposed technology or solution.
- Effects: Potential benefits and harms arising from the development and implementation of the technology or solution.
- Values: Examination of the most pertinent ethical considerations related to the effects from step II.

Stage 3: Action points

Action points to promote responsible innovation at 3 levels of abstraction:

- Technology*:* How should it be designed?
- Environment: Includes both tangible elements (eg, physical infrastructure) and systematic aspects (eg, policy).
- User: What can users do?

#### Participant Recruitment

Recruitment took place within the Dutch health care and research context through 2 structured routes. First, invitations were distributed via the LEAPfROG consortium network, including affiliated partners and organizations, following the project’s progress. Second, patient participants were recruited through the Dutch Kidney Patients Association (Nederlandse Vereniging voor Nierpatiënten [NVN]). When invitees were unavailable, comparable participants were identified outside these routes to maintain diversity across stakeholder groups. All invited participants received a written background document explaining the LEAPfROG project, the purpose of the workshops, and their expected contribution. No prior preparation was required for participation. Patients were invited as experiential experts rather than professionals and were offered an introductory meeting to ask questions and familiarize themselves with the process. All participants provided informed consent before the workshops took place.

Before the workshops, participants were notified of the intention to publish a report on the session results and were given the opportunity to raise objections. Prior to publication, they were able to request changes and provide consent for the final reports, which are available in Dutch as MultimediaAppendices 2  3. The first workshop, led by external moderators from Stichting ECP, was not audio-recorded. To further strengthen the accuracy and completeness of the documentation, the second workshop was audio recorded, with participants informed in advance and given the opportunity to object.

#### Documentation and Validation

In both workshops, discussions were documented on flip charts or a shared screen, allowing participants to review and refine notes in real time. Notes from moderators and researchers were later compared and synthesized into validated reports. The GEA emphasizes collective ethical reflection, in which differing perspectives are explicitly explored rather than reconciled into consensus. In the first workshop, notes were taken independently by 2 external moderators from Stichting ECP, alongside 2 researchers (JEK and MTM). The moderators’ notes from the first session formed the basis for the initial draft of the first workshop report. Notes from JEK and MTM were compared, reconciled, and extensively discussed within the research team to ensure completeness and accuracy. This iterative process led to the final validated report. The first draft of the second workshop report was prepared by MTM and subsequently finalized through joint review by MTM, JEK, and JEL.

#### Workshop Data Analysis

The workshop discussions, facilitator notes, and summary reports were thematically analyzed using the GEA framework, which emphasizes dialogue and collective reflection among diverse stakeholders. The analysis focused on how participants articulated ethical and practical considerations across the 3 reflective phases of the GEA: mapping the technology in context, ethical reflection, and translation to action. MTM led the review and synthesis of the workshop materials, identifying recurring themes and insights that emerged through these dialogues. The resulting reports were designed as standalone outputs for dissemination within the LEAPfROG consortium. For the purpose of this paper, the analysis focused on themes most relevant to the research gap at the intersection of EHR data and AI development. The coding framework developed from the scoping review provided the analytical foundation, guiding the thematic structure while allowing refinement and contextualization based on stakeholder perspectives. Themes were subsequently reviewed and refined with the coauthors during manuscript preparation to ensure consistency and alignment with the broader study.

### Ethical Considerations

The overall LEAPfROG project protocol was reviewed by the Medical Ethics Committee of Amsterdam UMC (the Netherlands). The committee granted a waiver of formal approval (W22_340 #22.412) because the LEAPfROG project, including all substudies, does not fall within the scope of the Dutch Medical Research Involving Human Subjects Act (WMO). All workshop participants were informed about the study objectives and procedures and provided informed consent to participate. Participation was voluntary and unpaid. Travel expenses were reimbursed where applicable. Participants were informed that workshop reports would be made publicly available, and any attribution of names or roles in those reports occurred with participants’ knowledge and consent.

## Results

### Included Literature

**Figure 1.**
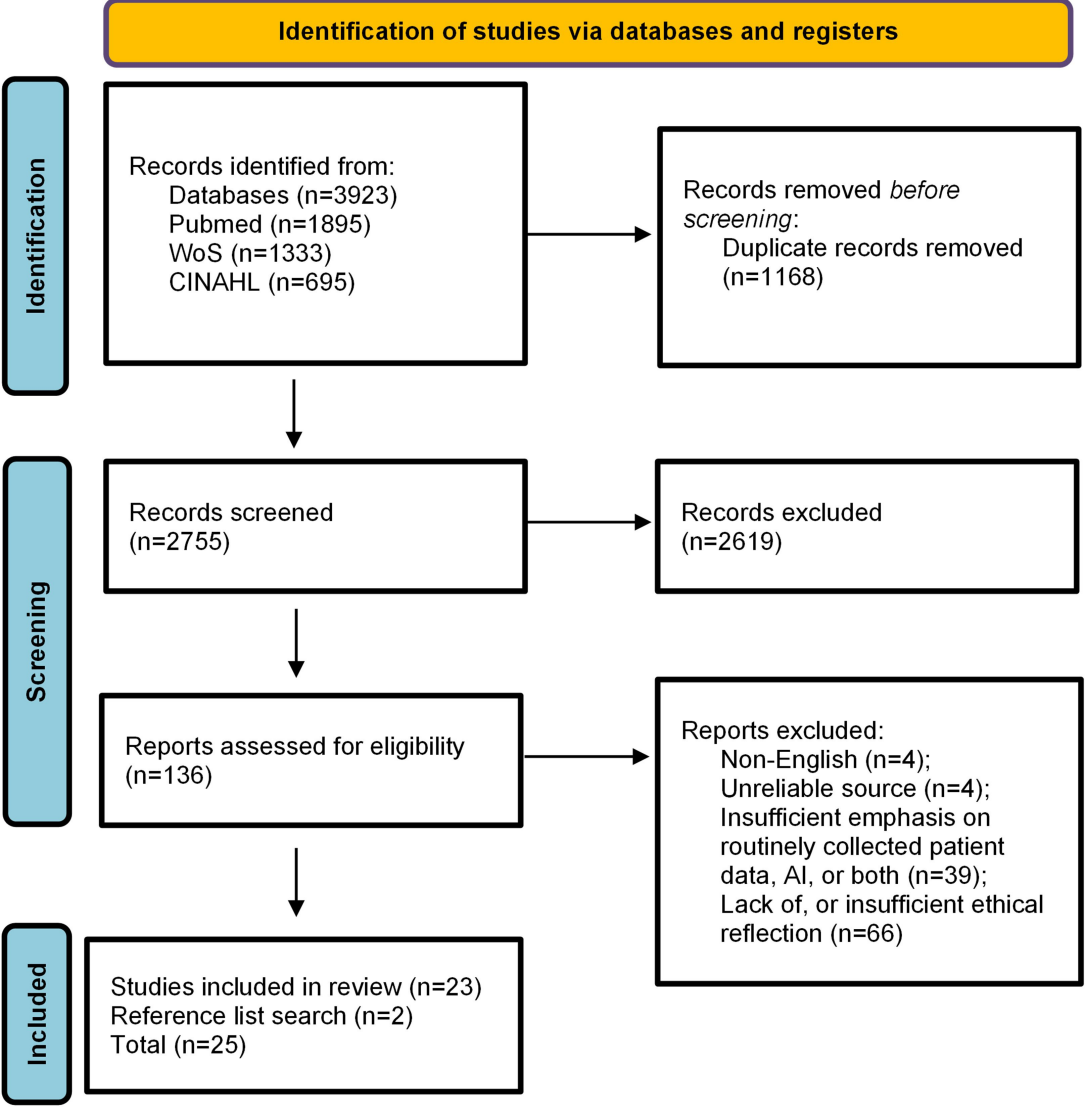
PRISMA (Preferred Reporting Items for Systematic Reviews and Meta-Analysis) flow diagram of study selection. AI: artificial intelligence.

The results of the literature screening are presented in a PRISMA flow diagram (Figure 1). In total, 25 articles met the inclusion criteria. Most were commentary-style publications (eg, perspectives and debates; n=14), alongside review-type articles (n=8) and a smaller number of qualitative studies (n=3). In terms of medical focus, the largest group of articles explored themes within general health care and clinical informatics (n=9). Other represented areas included medical ethics and health equity (n=5), primary care (n=3), public health and health disparities (n=2), geriatrics (n=1), psychiatry (n=1), nephrology (n=1), rheumatology (n=1), pediatrics (n=1), and orthopedics (n=1).

In terms of geographical distribution, the majority of articles were published by authors based in North America (n=14), followed by Europe (n=8), Asia (n=2), and Australia (n=1). A detailed overview of article characteristics is available in Multimedia Appendix 4 [22-46].

### Workshop Participants

Two stakeholder workshops were held, with 17 participants in the first and 13 in the second, with some attending both. Participants included medical ethicists (n=3), medical informatics experts and AI engineers (n=9), patients associated with the Kidney Patients Association Netherlands (NVN) (n=2), patient representatives from the NVN (n=2), a nephrologist, a chief medical information officer (n=1), a chief scientific information officer and endocrinologist (n=1), a general practitioner (n=1), pharmacists (n=2), a digital transition manager, data protection officers (n=2), and a digital ethics manager.

### Key Themes From the Literature and Integration of Stakeholder Workshops

This section is structured around 4 key themes: data privacy, transparency, and consent; public trust and regulatory challenges; fair representation and model generalizability; and responsible AI integration in clinical practice. This structure reflects the staged design of the LEAPfROG project and the cyclical nature of AI development in health care, which typically extends across data governance and quality, model generalizability, and clinical implementation, with each dimension influencing and being influenced by the others across policy, data, AI, and practice. The themes raised during the second stakeholder session aligned with the coding scheme developed from the scoping review, reinforcing the relevance of the literature to stakeholder perspectives. The workshops played a crucial role in grounding the analysis in real-world experience, ensuring its practical relevance within the LEAPfROG project.

To illustrate how thematic emphasis shifted across the different study components, Table 1 presents the staged development of these themes across the scoping review and stakeholder workshops.

**Table 1.** Shifts in thematic emphasis across study components of the staged LEAPfROG (Leveraging Real-World Data to Optimize Pharmacotherapy Outcomes in Multimorbid Patients Using Machine Learning and Knowledge Representation Methods) design.

Key themes / stage	Workshop 1 (EHR^a^ data reuse)	Scoping review (EHR–AI^b^ Integration)	Workshop 2 (AI development and implementation)
1. Data privacy, transparency, and consent	Patient autonomy and pseudonymization challenges Clear consent for secondary use of EHR data	Privacy risks amplified by data linkage and free-text inputs; persistent reidentification vulnerabilities Tensions between ethically robust consent and notions of a civic duty to share EHR data	Fostering trust through transparency across AI model development, explainability, and outputs
2. Public trust and regulatory challenges	Institutional fragmentation and suboptimal national EHR infrastructure Inconsistent policy and legal interpretations among stakeholders (including data custodians and researchers)	Siloed governance and fragmented oversight Regulatory uncertainty limiting data sharing and accountability	Stakeholder-led, value-driven governance Shared vision on responsible EHR data reuse and multistakeholder collaboration
3. Fair representation and model generalizability	Data heterogeneity and variability in EHR documentation practices Digital and social inequities shaping clinicians’ use of EHRs and patients’ access to their health data	Missing or biased data reinforcing inequities Lack of demographic diversity and transparency in reporting	Variation in clinical practices (eg, workflow diversity) shaping EHR data patterns and subsequent model outputs and interpretation Need to recontextualize EHR-derived data across hospital departments and care settings
4. AI integration in clinical practice	Documentation burden and workflow diversity in CKD^c^/DAKI^d^ care Impact on EHR data reliability and clinician workload	Model opacity, “clinical tropism” Risk of overreliance and clinician deskilling Ethical need for interpretability and accountability	Timing and interpretability of AI model outputs Risk of overly general or narrow outputs with limited clinical relevance Model could enhance clinicians’ understanding of drug side effects and support shared decision-making

a EHR: electronic health record.

b AI: artificial intelligence.

c CKD: chronic kidney disease.

d DAKI: drug-induced acute kidney injury.

We connected insights from the literature and stakeholder discussions to situate the key themes within the Dutch health care landscape and the specific context of the LEAPfROG project. Each of the following sections elaborates on one of the 4 key themes and concludes with a reflection on how that theme was grounded and contextualized through stakeholder discussions.

### Data Privacy, Transparency, and Consent

#### Privacy Risks and Security Challenges

The reviewed literature highlights growing privacy concerns around the reuse of EHR data for AI research [22-34]. EHR data originate from everyday clinical care and often contain sensitive personal and medical information recorded without explicit patient consent, raising concerns about misuse and breaches of confidentiality [30]. In contrast, data from controlled research settings such as randomized trials follow predefined consent procedures and strict regulatory oversight.

Safeguarding privacy in this context requires clear data access agreements, secure systems for storage and transmission, and transparent mechanisms for patient control and consent, in line with legal and ethical standards. The risks described in the literature include security breaches, such as hacking or malware attacks, that could lead to data exploitation, intentional discrimination, or identity theft [27,31,35]. Additionally, combining multiple data sources, such as EHRs and disease registries, amplifies privacy risks through greater exposure to breaches, inconsistencies, and loss of data integrity [23,27,28,30,34,36]. Advances in AI have further intensified the risk of misuse and harm by enabling sophisticated reidentification, tracking, and profiling techniques that threaten patient confidentiality, including methods capable of inferring sensitive information from nonsensitive data. These developments heighten the risk of misuse and harm [28,35]. Even privacy-preserving approaches remain vulnerable to inference attacks that can reveal attributes of training data, posing ongoing risks to confidentiality [35].

Moreover, free text fields in EHRs often include both medical and personal information. Persisting difficulties in deidentifying this information increase the potential for reidentification and unintended disclosure when used for research purposes, possibly even beyond the individual patient [27]. Ford et al [27] discuss the challenge of balancing automated and human-led deidentification of free text, noting that participants in citizen jury discussions expressed concerns that automated methods might leave identifiers, while human-led processes, on the other hand, could introduce bias or error due to inconsistency or workload. Others emphasize that the use of AI methods to extract information from unstructured EHR text often lacks standardized guidelines and the level of scrutiny applied to other medical interventions, further exacerbating privacy risks [33].

#### Informed Consent: Is There a Civic Duty to Share Data for AI Health Research?

Most included studies identify challenges related to the need for explicit patient consent in reusing EHR data for AI-driven research [23,25,27-31,33-35,37,38]. Patients are often unaware of how their data may be used over time, raising questions about the validity of prior or implicit consent given for clinical care or research. Evolving AI models may introduce new applications that extend beyond the original scope of consent, repurposing data in ways that patients could not have anticipated [31,38]. These concerns intensify when multiple data sources are linked, making it difficult to ensure meaningful consent at each stage of data integration [22,23,38,39]. Without clear processes for obtaining and maintaining consent, AI-driven research risks undermining patient autonomy and trust in health data governance, compromising its moral legitimacy.

Among the included studies, Müller critically examines the view that citizens have a moral duty to share their health data for medical AI development. He argues against this by outlining key contrasts between common assumptions underlying such a duty and the ethical realities of data sharing [28]. First, the “rule to rescue,” which asserts a duty to help others in immediate danger, does not apply here, as data sharing lacks the direct, person-to-person moral obligation implied in such situations. Second, the “low risks, high benefits” argument claims that societal gains justify data sharing. He argues that this view overlooks how risks such as privacy breaches and erosion of trust in health care are unequally distributed and more immediate than potential benefits. Third, the “property rights argument” holds that the involvement of public institutions in generating health data justifies limiting individual ownership. Müller disputes this, emphasizing that health data are inherently personal and sensitive, and that treating them as a public commodity undermines autonomy and confidentiality.

Instead of a universal moral duty, Müller proposes a civic responsibility approach grounded in transparency, value alignment, and voluntary participation, emphasizing consent mechanisms that allow individuals to manage how their data are shared. In line with this perspective, other authors argue that even when data reuse does not legally require consent, researchers remain ethically obligated to communicate clearly how data are used, how AI models operate, and how decisions are reached, as legal compliance does not substitute for transparency or patient and public engagement [33].

#### Stakeholder Perspectives: Data Privacy, Transparency, and Consent

In line with the literature, the stakeholder group discussed potential risks to patient privacy in the reuse of EHR data for research. They identified data exchange and EHR systems linkages as particularly vulnerable points, along with the risk of unauthorized data use by third parties such as health care insurers and private companies. To address these concerns, stakeholders proposed several measures, including the use of virtual research data environments that strictly comply with data protection regulations, ensuring that research data are pseudonymized or anonymized to reduce the risk of misuse or breaches, and enabling data providers to audit researchers’ actions on the data.

Other suggestions involved giving patients greater control over how their EHR data is reused, including the ability to decide whether, and which parts of, their records should be used for research. While this approach could enhance patient autonomy, concerns were raised about the risk of overburdening patients with repeated consent requests, especially when the research offers no clear or direct benefit at the individual level and does not immediately reflect patient priorities.

### Public Trust and Regulatory Challenges

#### Trust and transparency

The majority of the studies highlight trust as crucial in the relationship between various publics and those who collect, control, access, and use EHR data for AI research [23-25,27,29,31-36,38,40-42]. Many studies identify low public awareness as a key factor undermining trust in these practices [24,27,32,36]. Patients are often unaware that their data are shared across organizations within integrated systems or for what specific purposes [31]. Low public trust in institutions handling health data may reduce participation in AI research, ultimately introducing self-selection bias and weakening model quality and representativeness [28,31,34]. Given these concerns, many studies underscore the ethical importance of transparency, not only as a means to foster trustworthiness but also for ensuring legitimate and accountable data governance [22,24,25,27-34,38,40,41].

#### Regulatory fragmentation and legal uncertainty

Several studies identify regulatory and legal uncertainties as major obstacles to the adoption of health AI tools [30,41,42]. A fragmented legal landscape, marked by inconsistencies across institutions and jurisdictions, creates compliance and accountability challenges [29,31,33]. In Germany, for example, data-sharing initiatives are complicated by regulatory fragmentation and varying requirements across sectors [29]. Siloed regulation, in which separate and uncoordinated frameworks govern different types of health data and medical technologies, fragments oversight, leading to regulatory gaps (areas not clearly covered by any authority), overlaps (conflicting or duplicated requirements), and broader compliance hurdles that hinder implementation [33,38]. Regulatory uncertainty often deters data custodians—responsible for data storage, integrity, and authorized access—from sharing EHR data beyond clinical settings. This reflects a broader tension between oversight predominantly aimed at safeguarding privacy and fostering the flexibility required for AI innovation. For instance, strict interpretations of data protection laws, such as the General Data Protection Regulation, can limit researchers’ access to patient data, influencing the trajectory of developments in the field [30,38].

#### Stakeholder Workshops: Public Trust and Regulatory Challenges

In the Netherlands, EHR data exchange remains limited due to the fragmented health care system, where institutions rely on diverse and often incompatible standards. For example, hospitals may use different clinical coding systems or inconsistent data formats for diagnoses, making integration across systems technically and semantically challenging. This challenge is further compounded by a decentralized governance framework that grants considerable institutional autonomy, resulting in varied requirements and interpretations across institutional policies. In line with the literature, stakeholders noted that unclear and inconsistent interpretations of laws and regulations create barriers to collaboration within a complex network of actors, including hospitals, health insurers, data vendors, EHR system providers, and research funders, each imposing specific conditions for data availability and reuse.

A key suggestion that emerged during the workshops was to develop a shared vision within collaborative networks on the “why” of reusing EHR data for research. This could be achieved, for example, through consensus workshops involving patients, health care providers, researchers, data managers, and data protection officers, with attention to values such as trustworthiness, solidarity, quality of care, and privacy. Participants also emphasized the importance of establishing shared agreements within these networks on which data should be recorded in the EHR, using predefined terminologies, and defining how and where these should be implemented.

### Fair Representation and Model Generalizability

#### Health Inequities Reflected in the Data

Most included studies warn that missing or incomplete patient data can compromise model performance and perpetuate existing health care inequities, disproportionately affecting marginalized groups [22-25,28,30,31,33,37,39-46]. These inequities often stem from social determinants of health, such as financial barriers, limited digital and health literacy, and structural marginalization. Failing to adequately consider these factors can result in flawed models that underestimate disease burden for certain groups and undermine the equitable distribution of benefits [25,36,37,39,42-44]. Several studies further highlight the need to account for the historical context of research on race and ethnicity, ancestry, and sex and gender minorities, particularly in the United States, where past exploitation contributes to ongoing distrust in health care systems [40,42].

#### Institutional and Structural Variability in Data and Its Impact on AI Data Quality

Several studies highlight how institution-specific practices, norms, and patient populations shape the composition of EHR data [22,24,25,28-33,35,37,40,42-46]. Factors such as patient proximity to clinics, visit frequency, insurance coverage, and differences in clinical protocols and diagnostic resources influence data representation [22,24,25]. For example, some institutions may request diagnostic tests earlier [30] or use activity-based financing, leading to an overrepresentation of codes tied to higher reimbursement, reflecting billing priorities rather than actual clinical complexity [22,25]. Sampling bias can occur when chronically ill patients, who interact more frequently with the health care system, are overrepresented, skewing patterns that do not reflect the broader population. Within institutions, departmental priorities and documentation practices may differ, contributing to inconsistencies in data capture [25]. Increasing reliance on data-intensive health care can reduce direct communication between medical staff, heightening the risk of incomplete or selective documentation [24].

While standardizing EHR data through structured formats and predefined categories may reduce inconsistencies, it also risks removing essential clinical context [27,34,36,44]. Excluding physicians’ notes and clinical judgments, for example, may lead to the loss of valuable information such as symptom descriptions, diagnostic reasoning, and the context preceding symptoms. This increases the likelihood of misinterpretation by researchers and AI models, potentially resulting in biased or unsafe decisions [27,30,36,44,46]. Yet, as Knevel and Liao [30] note, some gaps in EHR data can hold clinical meaning. For instance, the absence of specific test results may suggest that a particular diagnosis was deemed unlikely. Interpreting such nuances requires strong domain knowledge and clinician involvement to ensure meaningful analysis.

Variability in EHR systems across institutions and countries continues to challenge the development of generalizable AI models [30]. The absence of centralized or standardized data-sharing mechanisms, such as those based on findability, accessibility, interoperability, and reusability principles, exacerbates this problem by hindering interoperability and seamless data integration across settings [23,33,34,36,38]. To address structural and institutional fragmentation, federated learning has been proposed as a promising approach aimed at preserving patient privacy while enabling AI models to be trained across institutions without sharing raw data. Such methods seek to facilitate collaboration across decentralized datasets while maintaining institutional autonomy. Related privacy-preserving techniques include differential privacy and homomorphic encryption, which are designed to enhance data protection but remain largely experimental and face challenges of scalability, performance, and validation in clinical settings [35].

#### Challenges in AI Model Reliability and Potential Harm

Several studies raise concerns about harm from unreliable or biased AI systems, particularly when embedded in clinical decision-making. Deliberate and unintentional choices in AI development can introduce algorithmic bias, reinforcing EHR data limitations and distorting model outputs [22,24,25,27,28,30-33,35,37,42,43,45]. Examples include data integrity issues from reusing and merging databases, such as data duplication [24,35], model design choices reflecting provider bias [32], and preselection of training data based on physician or developer preferences [28,46].

Technical flaws throughout model development can also result in harmful clinical outcomes, even when using high-quality data [33,37,43]. For example, models may overfit datasets that do not reflect relevant clinical questions, undermining generalizability and reliability [33,37,43]. Optimistic predictions can lead to futile or even harmful interventions, while overly pessimistic ones may cause unnecessary withholding of care [32]. In such cases, the ethical imperative to minimize harm may justify simpler models that generalize more safely, even if less accurate. Some studies argue that the appropriate level of model explainability should be calibrated according to the clinical risks and the extent to which domain experts can interpret the model’s output [24,33,37,46]. Several studies also highlight the lack of standardized frameworks or tools to assess and evaluate AI’s real-world clinical impact, particularly regarding patient safety, value alignment, and health equity, thereby limiting consistent assessment of ethical implications and potential harm [25,26,29-34,38,40,43-45].

#### Barriers to Inclusive AI Development

Several studies identify deeper systemic and infrastructural barriers that undermine inclusive and equitable AI. Adapting AI to local environments requires significant investment in data integration, testing, infrastructure, and clinical expertise [22,30]. However, many health organizations lack the secure, scalable infrastructure needed for effective AI deployment, a gap often overlooked in policies that emphasize AI’s benefits while downplaying resource demands [22]. These barriers also raise questions of equity in data governance, including whether and how data contributors should be compensated or share in AI’s benefits [31].

Another persistent challenge is the unclear definition of target populations and the inconsistent representation of demographic groups in AI development [22,25,28,41,45]. One study found, for example, inconsistent demographic reporting across 164 EHR-based AI studies, undermining model representativeness and reproducibility [45]. Limited data sharing, closed-source models, and poor interoperability hinder validation across diverse clinical settings, while low digital adoption and data vendor monopolies further restrict datasets to less representative populations [25,28,29,32-34,36,38,41,42,45].

Paulus and Kent [42] argue that fairness in algorithmic decision-making is context-dependent, requiring value judgments and stakeholder consensus. No universal definition exists, and ethical concerns over protected attributes such as race persist. Others similarly highlight concerns about the values and priorities embedded in AI models, which may implicitly reflect biases or unexamined assumptions [44]. This risk intensifies when health data is used for generating profit, for example, potentially placing financial incentives above equitable patient care and responsible data stewardship [30,31,33,34]. In response, many studies advocate for sustained stakeholder engagement throughout AI development efforts, including participatory co-design and mixed methods approaches that integrate qualitative and quantitative insights rather than relying on externally imposed solutions [22-25,27,28,32,33,37,40-44,46].

#### Stakeholder Workshops: Fair Representation and Model Generalizability

Stakeholder discussions highlighted the complex realities of clinical practice in the context of CKD and DAKI, which are inherently mirrored in the data. For instance, when treating sepsis with antibiotics such as vancomycin, known to potentially cause acute kidney injury (AKI), clinicians may consciously accept the risk of AKI as a necessary trade-off to, in some cases, save the patient’s life. Additionally, when a side effect is common and widely acknowledged, physicians may not document or provide less detailed documentation, focusing instead on more pressing clinical problems. Moreover, these considerations can also vary across medical specialties, with clinical practices such as prescribing practices differing, for example, between internal medicine and cardiology.

In short, understanding a potential causal relationship between a drug and AKI provides only part of the story. During the workshops, it was emphasized that gaining a clear understanding of how variability from different clinical settings is represented in the EHR data is crucial, as is considering how such complexity could be effectively incorporated into an AI-driven decision-support model for retrospective DAKI diagnosis. At the same time, consistent with Knevel and Liao [30], it was noted that research using EHR data can offer valuable insights into clinical routines and decision-making patterns, such as differences in prescribing practices across specialties. However, uncovering these insights is highly resource-intensive and time-consuming, as it requires not only technical expertise but also deep contextual knowledge and sustained engagement with clinical practice.

### Responsible AI Integration in Clinical Practice

#### Administrative Workload

While efforts to structure and standardize EHR data are central to enabling AI-driven research, several studies emphasize the unintended increase in administrative burden for health care providers [24,33,35-38,40,41,44]. Processes such as careful data assessment and logging, essential for ensuring data quality, reducing fragmentation, and addressing issues like concept drift in longitudinal datasets, are time-consuming and resource-intensive [33,37,41]. These demands raise ethical concerns related to clinician well-being, sustainable work practices, and their impact on patient care [24,29,36,41]. Furthermore, several studies note that professionals’ earlier experiences with EHR implementations often failed to deliver the anticipated quality improvements despite significant increases in workload associated with data entry and system management, contributing to skepticism toward data-driven AI solutions [24,29,41]. Finally, many health care settings still lack fully digitized, structured records, complicating the integration of AI into clinical workflows [41]. Together, these challenges highlight a tension between the technical demands of fit-for-purpose data in AI-based research and the practical realities of clinical work, underscoring the need for implementation strategies that balance data quality with provider sustainability.

#### Clinical Tropism

A major barrier to AI adoption in health care is the limited transparency and interpretability of complex, “black-box,” models [22,24-28,30-34,36-41,43-46]. When AI-driven decisions lack clear explanations, clinicians and patients struggle to trust them, undermining ethical requirements for transparency, informed decision-making, and accountability in clinical care. Without insight into how these models function, users cannot detect or correct biases, increasing the risk of erroneous or harmful outcomes [22,39,41]. Distinguishing causal from correlative relationships is essential in medical decision-making to avoid ineffective or harmful interventions [28,39]. These risks are further amplified by the fact that harmful AI-related errors can occur at scale, exceeding the impact of individual provider errors [22].

As discussed earlier, many studies highlight challenges of representativeness and model generalizability, emphasizing how incomplete or biased datasets can perpetuate existing inequalities. Beyond these population-level concerns, several studies draw attention to how AI systems mirror the clinical contexts in which they are developed. Alami et al [22] describe this as clinical tropism, referring to the tendency of AI systems to reproduce the specific practices, routines, and priorities of the settings that generated their training data. They argue that models trained on localized data may reflect institutional protocols, workflows, or device infrastructures that are not easily transferable to other contexts. Over time, such systems risk becoming repositories of established clinical practices, developing what Alami et al [22] term a “clinical mind,” rather than generating new insights. This dynamic may lead professionals to place disproportionate trust in AI outputs that merely echo existing patterns of care. Furthermore, because clinical practice continually evolves through new treatments and protocol updates, EHRs are inherently temporal, making input data prone to diverge from the contexts represented in their underlying sources [22].

#### Overreliance and the Risk of Deskilling

Several studies highlight the risk of deskilling among medical professionals as AI adoption increases, potentially weakening clinical judgment, autonomy, and the ability to make independent decisions [22,25,26,29-32,37-39,41,43]. Overreliance on AI may also reduce adaptability across clinical contexts and limit clinicians’ capacity to account for patient preferences and values, raising concerns about the long-term impact on clinical competency and patient care [22,25,26,32,37,43].

Empirical evidence suggests that caregivers (including nurses, midwives, clinicians, and pharmacists) are generally less likely to question algorithm-driven diagnostic results, increasing the error risk and further eroding clinical judgment over time [22,25,28,37]. Conversely, when AI-generated results deviate considerably from a clinician’s assessment, there is a risk that the model’s recommendations will be dismissed outright, potentially limiting AI’s practical value in clinical settings [22,43]. These dynamics underscore the need for careful integration of AI into clinical workflows, with sustained attention to its effects on professional roles, decision-making, and oversight.

#### Stakeholder Workshops: Responsible AI Integration in Clinical Practice

In the complex care setting of CKD and DAKI, precise documentation of medication use and adverse drug events is essential for generating reliable data to support AI-driven research. However, DAKI is difficult to diagnose because of its multifactorial causes, subtle onset, and patient-specific variability, often involving combinations of dehydration, sepsis, and drug toxicity. During stakeholder workshops, participants stressed that documenting this complexity is time-consuming. One general practitioner noted that recording side effects alone can take up to 30 minutes, a significant burden given current time pressures in Dutch health care. Although general practitioners are legally required to provide digital access to medical records upon request, they are not obliged to use an EHR system. One kidney patient shared that her general practitioner still prefers pen and paper, relying on digital systems only when necessary, illustrating how variability in documentation practices continues to limit the availability of structured data for AI.

Participants saw potential for the model to have a learning effect, improving clinicians’ understanding of drug side effects over time. This could enhance care quality and, if communicated clearly, support shared decision-making. However, they stressed that model outputs must always be evaluated alongside clinical expertise to ensure safe and appropriate care. To improve clinical relevance, they suggested linking outputs to relevant guidelines and scientific literature to help physicians interpret findings in context and assess their significance for individual patients. At the same time, they cautioned that the time required to interpret outputs might increase or shift clinical workload rather than reduce it.

Stakeholders raised concerns consistent with the concept of “clinical tropism” of Alami et al [22], which describes how AI models may reproduce the narrow or context-specific knowledge embedded in the data on which they are trained. They noted that this tendency could be reinforced by the steering effect of clinical guidelines and institutional protocols that shape both data collection and model performance. Participants further noted that publication bias in the studies informing model development can compound these effects. When only positive or statistically significant results are published, the evidence base fails to reflect the full range of clinical outcomes, leading models to learn a distorted view of clinical reality, thereby reinforcing rather than mitigating clinical tropism. Together, publication bias and the standardizing influence of clinical guidelines narrow the evidentiary foundation on which AI systems are built, privileging typical or well-studied cases while obscuring clinical variability and local practice. Participants also cautioned that integrating diverse data sources may produce overly general outputs with limited clinical relevance, lacking actionable value for clinicians and therefore likely to be disregarded.

Finally, participants stressed that assessing the causes of AKI requires attention to factors beyond medications and careful interpretation of predicted probabilities within individual patient contexts. For instance, a 61% probability that an antibiotic caused AKI might be clinically insignificant in one case but highly relevant in another, depending on the patient’s circumstances. Participants emphasized that the model should be developed and implemented primarily as a decision-support tool to help clinicians assess whether a drug likely contributed to AKI.

## Discussion

### Principal Findings

This study used a mixed methods approach to explore the ethical considerations of using EHR data for AI-driven health research and innovation, with the LEAPfROG project serving as a guiding case. We combined a scoping literature review, a systematic search, and 2 stakeholder engagement workshops, all informed by the GEA [17].

Previous studies have examined the reuse of EHRs and the ethical implications of AI development separately. Research on data reuse has addressed governance, consent, and public trust, showing how challenges of transparency, fairness, and oversight are amplified by the data’s variable quality, uncertain provenance, and fragmented governance [12-14]. Studies on medical AI have focused on fairness, transparency, and accountability, mapping the ethical and epistemic risks that arise within algorithmic systems [15,16]. Our study brings these strands together by examining how recurring ethical challenges emerge and take shape at the intersection of EHR data and AI development, while also situating these issues within a real-world clinical and institutional setting (the LEAPfROG project).

By integrating stakeholder perspectives with existing ethical analyses, this study contributes an empirically grounded account of how recurring ethical challenges emerge in practice when AI systems are developed using EHR data. In this context, issues of consent, privacy, and governance become more complex as EHR data is repurposed and leveraged using AI, while the opacity and scale of AI systems introduce new risks of decontextualization and fragmented accountability across policy and oversight domains.

Building on this analysis, we advocate for an approach to data governance and AI development that is centered on and led by stakeholders. We stress the importance of involving all relevant parties not only in evaluating benefits and risks, but also in shaping the goals and direction of AI innovation. Accordingly, we structure the following discussion as follows: first, we present a set of practical recommendations derived from the literature and stakeholder workshops, intended to guide future reflection, engagement, and implementation. We then explore the concept of data work and examine the ethical risks associated with removing data from its original context and reusing it for AI development. Finally, we review current practices in stakeholder engagement and highlight the need for more inclusive and participatory forms of governance in future AI initiatives.

### Practical Recommendations

In addition to the central themes, both the scoping review and stakeholder workshops yielded practical recommendations for addressing ethical concerns related to AI and the use of EHRs (Table 2). Although these action points were not the primary focus of this study, they offer opportunities and potential entry points for addressing the broader challenges we aimed to explore. These insights reflect the perspectives and priorities identified in the sources consulted and do not necessarily represent the position of the author group. As the findings draw on international literature, some recommendations, such as patient representation in ethics committees, may already be established in contexts like the Netherlands. Nevertheless, they offer a useful supplement for future reflection, engagement, and implementation.

**Table 2.** Key recommendations from literature and stakeholder workshops.

Key themes and recommendations	Main actors	Source (key articles)
Data privacy, transparency, and consent		
Strengthen AI^a^ and data literacy among patients, clinicians, and institutional review board (IRB) members, emphasizing how EHR^b^ quality and AI training may affect privacy, consent, and data integrity.	Board of Directors of health care organizations	[38,41]+ workshops
Implement dynamic consent mechanisms that reflect the iterative reuse of EHR data in AI development and ensure institutional governance structures can accommodate these updates.	Board of Directors of health care organizations	[27,28,31]
Integrate consent management tools into EHR interfaces so that patients can review and modify how their data is used in research and model training, with clinical staff facilitating informed communication.	Board of Directors of health care organizations and EHR system vendors	[25,31]+ workshops
Require transparent documentation of AI provenance, including funding sources, proprietary model components, and training data, to prevent hidden conflicts of interest and foster public trust.	Researchers involved in AI projects like the LEAPfROG^c^ project	[23,25,29]
Public trust and regulatory challenges		
Adopt standardized, FAIR-aligned^d^ data practices and establish cross-institutional agreements that clarify ownership, accountability, and data flow between hospitals and general practices.	National research infrastructure organizations like Health-RI in the Netherlands	[33,34,36,40]+ workshops
Establish joint oversight frameworks that include clinicians, data stewards, developers, and regulators to coordinate decision-making on data use, ownership, and third-party involvement, reducing fragmented accountability.	National research infrastructure organizations like Health-RI in the Netherlands	[23,25,29,31]+ workshops
Embed patient representatives (ideally trained in AI ethics and data governance) in ethics committees and regulatory boards to strengthen inclusiveness and transparency in oversight processes.	National research infrastructure organizations like Health-RI in the Netherlands and Board of Directors of health care organizations	[25,38,41].
Fair representation and model generalizability		
Validate AI models in real clinical environments (for example, CKD^e^ and AKI^f^ contexts) and across diverse patient subgroups to ensure representativeness and clinical relevance.	Researchers involved in AI projects like the LEAPfROG project	[37,42,43,46]
Conduct fairness audits to explicitly assess whether model outputs are biased by demographic differences or by specific clinical routines and documentation practices that vary across institutions.	Researchers involved in AI projects like the LEAPfROG project	[32,40,42,43]
Adopt mixed methods evaluation combining quantitative metrics with qualitative insights to understand how AI affects workflows, professional judgment, and patient experience.	Researchers involved in AI projects like the LEAPfROG project	[30,40,42,43]+ workshop 2
Implement continuous postdeployment monitoring: reassess clinical impact on patient outcomes over time to ensure alignment with evolving clinical practice and technologies.	Board of Directors of health care organizations	[25,38,46]+ workshop 2
Appraise both clinical and economic outcomes through frameworks that evaluate how AI contributes to care quality, efficiency, and sustainability of health care delivery.	Researchers involved in AI projects like the LEAPfROG project	[22,39,46]
Responsible AI integration in clinical practice		
Align AI development with clinical workflows, prioritizing usability and practical relevance over purely technical performance metrics.	Researchers involved in AI projects like the LEAPfROG project	[22,24]+ workshops
Ensure interpretability supports clinical judgment by designing models that reflect clinical complexity, communicate uncertainty, and complement human expertise.	Researchers involved in AI projects like the LEAPfROG project	[22,26,37]+ workshop 2
Develop learning-oriented AI systems that enhance, rather than erode, professional expertise, and support reflective clinical practice.	Researchers involved in AI projects like the LEAPfROG project	Workshop 2
Design EHR/AI tools collaboratively with stakeholders throughout the data and model lifecycle to clarify purposes, risks, and benefits, aligning these with shared values and patient expectations.	Researchers involved in AI projects like the LEAPfROG project and Board of Directors of health care organizations	[25,33,38,41]+ workshop 1

a AI: artificial intelligence.

b EHR: electronic health record.

c LEAPfROG: Leveraging Real-World Data to Optimize Pharmacotherapy Outcomes in Multimorbid Patients Using Machine Learning and Knowledge Representation Methods.

d FAIR: findability, accessibility, interoperability, and reusability.

e CKD: chronic kidney disease.

f AKI: acute kidney injury.

### Making Data Work

While much of the public and academic debate on AI in health care focuses on model explainability or algorithmic opacity, these discussions often overlook a more foundational issue: the nature of the data on which AI depends. Earlier studies on health data reuse have shown [12-14] that questions of trust, consent, and governance already hinge on how data is produced, managed, and interpreted. Our findings suggest that without a deeper understanding of how health data is actually generated, structured, and used in practice, efforts to build fair, accurate, and trustworthy AI systems will remain incomplete. Data is rarely a neutral input, as it is deeply shaped by clinical routines, institutional diversity, and social context. Documentation practices, system design, and socio-ethical expectations determine what gets recorded and how it is organized. Recognizing this “data work” is not merely a technical concern but an ethical one, since it determines whose experiences are recorded and how decisions are justified.

Our findings reveal the complexity and effort involved in making EHR data fit for AI-driven research. Both the reviewed literature and stakeholder insights point to inconsistencies in EHR documentation, variability in clinical workflows, and the labor-intensive nature of data preparation and standardization. Such challenges underscore that data is produced through ongoing, situated work rather than passively collected. This aligns with the literature review on data-related activities by Bertelsen et al [47], or “data work,” which emphasizes its multifaceted and situated nature. They group data work into 3 interrelated categories: data collection (eg, capturing, discovering, requesting, and self-tracking), data production (eg, coding, entry, and digitization), and data use and sharing (eg, providing, exchanging, and disseminating).

As data move through different stages and are reused for AI training, they often lose the clinical and institutional contexts that once gave them meaning. This process, referred to here as decontextualization, involves detaching data from the social and professional environments in which they were produced, allowing them to be reinterpreted, or even misinterpreted. Hoeyer [48] describes this as a separation between the “data work of production” and the “data work of analysis,” where separation from the original context renders data fragile and open to misinterpretation. In AI development, this dynamic has ethical as well as epistemic consequences: models may treat context-dependent judgments or incomplete entries as objective facts, obscuring the human and institutional processes that shaped them. This dynamic is also reflected in the concept of “broken data,” which highlights the ongoing, often hidden repair and improvisation required to keep data fit for purpose [49], as well as in studies showing how context loss invites interpretive uncertainty, with trust, visual design, and perceived credibility shaping how data is understood [50].

When AI systems trained on such data are applied in clinical settings, the same loss of context can reappear, shaping how models interact with the environments that produced their data and often reinforcing the very patterns they were meant to improve. Stakeholders noted that AI systems may mirror existing prescribing and documentation practices, reproducing the same routines they aim to change. This recursive dynamic reflects what emerged from the literature as clinical tropism, where models embed themselves in the patterns most strongly represented in their training data and in the settings where those patterns persist. When that influence begins to shape the outcomes it predicts, it can result in a self-fulfilling prophecy [51]. In such cases, models appear accurate precisely because their use helps make their predictions true. Clinical tropism creates the conditions, while the self-fulfilling prophecy describes the result. Together, they show how decontextualization can evolve from an issue within data to one that reshapes clinical practice, as models begin to reproduce the very patterns they were designed to improve. These concerns are mirrored in debates on explainable AI, where current approaches have been criticized for creating a false sense of understanding and trust, offering surface descriptions of model behavior without clarifying whether decisions are reasonable or justified [52]. Rather than mitigating bias, such transparency can obscure how context loss shapes both model development and evaluation.

Extending the discussion of decontextualization, Hoeyer [48] draws attention to a deeper issue often overlooked in discussions of data work: the ways in which data are interpreted and mobilized in pursuit of narratives that are often implicit and conflicting. He argues that datafication fragments patient information into pieces that can be recombined to serve diverse aims, such as clinical care, research, governance, or performance monitoring. In this process, the patient may be obscured, and data detached from the context of its production. Clinicians may document with specific knowledge and intent, while analysts or policymakers may act on institutional logics shaped by different priorities. These tensions are not resolved through technical solutions alone but often reappear, in new forms, within the very tools developed to address them. For instance, even privacy-preserving approaches such as federated learning, which are designed to mitigate data sharing risks, can inadvertently deepen decontextualization by abstracting data use from the institutional and clinical settings in which they originate [35,53]. By enabling model training across institutions without sharing raw data, these methods aim to enhance privacy but may nevertheless reproduce institutional differences and context loss, introducing additional ethical concerns that extend beyond the scope of this paper but are highlighted elsewhere [53].

As is evident in the LEAPfROG project, AI initiatives involving EHR data span a diverse network of stakeholders, including health care providers, data custodians, EHR vendors, patients, and institutions, whose interests may not align. These misalignments are not incidental but intrinsic to how data is leveraged, representing structural and arguably unavoidable realities that must be acknowledged. Developing responsible AI, therefore, requires collaborations that explicitly recognize these dynamics and openly address how stakeholders themselves navigate and negotiate data use in practice, while articulating the competing aims, values, and uses of data within their own processes [47]. Doing so requires more than technical expertise: it depends on collaboration across domains to understand how clinical, organizational, and social contexts shape data and influence AI training and deployment. Together, these insights underscore that making data work is as much a social and ethical practice as a technical one.

### Beyond Compliance: Meaningful Stakeholder Involvement

In current debates on health data governance, the public good is often invoked as a guiding principle, with values such as solidarity promoted as ethical foundations [54]. Prainsack et al [55], for instance, frame data solidarity as a means to promote justice, prevent harm, and ensure collective benefit. However, critics argue that such values, while well-intentioned, are often applied in top-down, unreflective ways that limit their ethical force [54,56,57] and risk overlooking deeper power asymmetries, such as the lack of patient representation in governance and decision-making structures. Rather than rejecting these principles, they have called for alternative models grounded in mutual aid and collective action, fostering more equitable relationships between data providers, users, and affected communities.

Taken together, these critiques reveal a broader structural issue: legal and policy alignment, while necessary, is not sufficient for the ethical use of health data or responsible AI development. As our results show, many studies point to the need for participatory, transparent governance that goes beyond compliance or rhetorical appeals to ethics. Concerns about fragmented oversight, unclear consent procedures, and opaque decision-making illustrate how legitimacy is undermined when stakeholders are not meaningfully involved. Without sustained, inclusive engagement, especially with those most directly affected, governance risks remain superficial and biased toward institutional priorities.

At a practical level, these structural shortcomings are reinforced by persistent power asymmetries in engagement processes, which risk prioritizing influential groups such as government officials, large nongovernmental organizations, or scientists [58]. These “elite” actors, with greater access to time, resources, and expertise, tend to set the terms of participation, sidelining less-resourced groups and limiting patient involvement. This is especially problematic in the context of CKD, where patients face complex treatments, uncertainty, and symptoms that can limit participation in research [59]. As a result, they are often underrepresented in studies that fail to reflect patient or caregiver priorities. Critical participation frameworks emphasize that such unequal conditions make engagement prone to tokenism, particularly when structural inequities like corporate influence or health care disparities remain unaddressed [59,60].

These dynamics become even more pronounced in the context of AI development. Here, technological optimism often frames innovation as inherently beneficial to patient care [61], which can obscure deeper ethical and social tensions. By emphasizing efficiency, accuracy, and progress, this framing tends to narrow debate to technical or policy issues, while sidelining questions of power and lived experience. Consequently, participation in AI development tends to be tightly controlled, serving institutional efficiency or public acceptance rather than fostering meaningful, value-driven dialogue. In this way, engagement risks becoming instrumental, legitimizing AI initiatives rather than engaging with lived experience or long-term ethical and practical implications.

In light of these challenges, we were particularly mindful of how stakeholder engagement was approached in the LEAPfROG workshops. Recognizing the potential influence of technological optimism, we sought to create space for more critical, grounded conversations about the role of AI in health care. By grounding ethical reflections in concrete use cases and fostering open-ended, collaborative discussions, we aimed to avoid tokenism and reduce the influence of more resourced stakeholders. Our mixed methods approach, which included a literature review alongside stakeholder workshops, helped broaden the scope to address systemic issues such as health care inequities and the resource-intensive nature of AI development.

As an alternative to top-down regulation and rhetorical appeals to ethics, more participatory and inclusive governance models may offer a promising path forward. One such approach involves shifting toward stakeholder-led governance within consortia, supported by coproduced guidelines or codes of conduct. A key suggestion from our workshops was to develop a shared vision around the reuse of EHR data, including its purpose, risks, and benefits, through collaborative processes involving patients, clinicians, researchers, data managers, and data protection officers. Such efforts could also include agreements on what data should be recorded in EHRs and how. Rather than treating participation as a means to secure legitimacy for predetermined goals, these practices aim to embed values like trustworthiness, solidarity, and privacy into the infrastructure of data governance itself. By continuing our collaboration with the Dutch Kidney Association (NVN), we hope to support a model of engagement that centers patient perspectives, addresses power imbalances, and ultimately aligns data-driven innovation with the needs of those most affected.

### Limitations

While time constraints, limited participant availability, and last-minute cancellations are common challenges in stakeholder workshops, a more critical limitation was ensuring balanced representation among stakeholders. Although the workshops were conducted in Dutch to promote inclusivity across diverse education levels and professional backgrounds, this choice inadvertently excluded non-Dutch-speaking participants, including some within the LEAPfROG consortium. Similarly, the scoping review included only English-language publications, potentially introducing language bias and omitting relevant studies in other languages. However, only 4 non-English publications were identified during screening, suggesting that such literature remains limited in this field.

In addition, the predominance of commentaries and reviews (14 out of 25) over empirical studies may limit the depth of evidence available to substantiate claims about real-world implementation, stakeholder experiences, or the practical implications. However, this imbalance also reflects the early and largely conceptual stage of ethical reflection at the intersection of AI and EHR data. Furthermore, many of the reviewed publications were authored by researchers without specialized legal or regulatory expertise, such as legal scholars deeply familiar with the GDPR (General Data Protection Regulation). This may have shaped how certain challenges were framed and further limited discussion of broader ethical issues, such as whether EHR data should be used for AI-driven research at all.

As mentioned above, our efforts to include diverse stakeholders may also have unintentionally reinforced power or knowledge imbalances, particularly due to the underrepresentation of patients. However, as described in the methods, patient participants were briefed in advance to support meaningful engagement. To enhance transparency and relevance, all workshop participants were also invited to review and comment on the workshop reports prior to publication. In addition, while policy perspectives were well represented in the workshops, participants with formal legal or regulatory expertise were underrepresented. This limitation may have influenced how governance and compliance challenges were discussed.

Furthermore, thematic convergence between the literature and the stakeholder workshop discussions may reflect the dominance of Global North perspectives, particularly those shaped by Dutch socioeconomic and institutional norms. While this alignment across methods strengthens the internal coherence of our findings, it also highlights a potential blind spot. Both the literature reviewed and the workshops conducted were situated within contexts that may not capture the unique challenges and priorities of the Global South. As others have noted, failing to account for these limitations risks overlooking perspectives that remain largely absent from this paper [62].

Beyond the institutional contexts examined here, large-scale health data initiatives (for example, the All of Us Research Program [63] and MIMIC-IV [64]) have likewise revealed persistent ethical challenges related to decontextualization, governance, and accountability. Similar questions are now emerging within Europe through the development of the European Health Data Space [54], which aims to harmonize secondary data use across member states. These examples demonstrate that the issues identified in this study re-emerge even in large-scale data initiatives with formal governance frameworks, suggesting that future research should further examine how context-specific governance practices can inform responsible data sharing at scale.

Empirical evaluation and the development of metrics to assess how the proposed ethical measures influence factors such as AI model fairness, data quality, or stakeholder trust fall outside the scope of this study. The goal of this work was to derive ethical measures through a novel approach that combines a scoping review with workshops based on the GEA. Future studies in other settings are needed to further develop and test evaluation frameworks that can operationalize these ethical measures in practice.

Our study deliberately focused on the Dutch and European legal and policy context to support LEAPfROG’s goals. The transferability of our stakeholder workshop methods to other countries or institutional settings was beyond its scope and could be explored in future empirical research. Future work could also broaden these discussions by addressing themes such as data sovereignty, the ethical dimensions of data labor in the Global South, and the potential for data colonialism [62,65].

### Conclusions

The use of EHR data in AI development is situated at the intersection of health care, data science, ethics, and policy, where competing priorities, values, and practices converge. Given this complex intersection, building responsible AI-based tools requires more than technical solutions and compliance. Our findings point to the need to begin with a critical understanding of the data, for which domain knowledge is essential. EHR data are not neutral but shaped by clinical routines, institutional constraints, and documentation practices. When removed from context, it is vulnerable to misinterpretation, which can cause AI systems to reproduce inequities and reinforce clinical patterns instead of supporting informed decision-making. Addressing these risks is essential to ensure accuracy and accountability. Our mixed methods approach, which included multistakeholder guidance ethics workshops, underscored the need for inclusive, value-sensitive development grounded in the realities of health care practice.

## Supplementary material

10.2196/79863 Multimedia Appendix 1 Search query details for systematic search.

10.2196/79863 Multimedia Appendix 2 Report of stakeholder workshop 1.

10.2196/79863 Multimedia Appendix 3 Report of stakeholder workshop 2.

10.2196/79863 Multimedia Appendix 4 Characteristics of included studies.

10.2196/79863 Checklist 1 PRISMA-ScR checklist.

## Abbreviations

AI: artificial intelligence
AKI: acute kidney injury
CKD: chronic kidney disease
DAKI: drug-induced acute kidney injury
EHR: electronic health record
GDPR: General Data Protection Regulation
GEA: Guidance Ethics Approach
LEAPfROG: Leveraging Real-World Data to Optimize Pharmacotherapy Outcomes in Multimorbid Patients Using Machine Learning and Knowledge Representation Methods
MeSH: Medical Subject Headings
NVN: Nierpatiënten Vereniging Nederland (Kidney Patients Association Netherlands)
PRISMA: Preferred Reporting Items for Systematic Reviews and Meta-Analysis
