# Supplementary material for: The Ethics of Leveraging Routinely Collected Patient Data for AI Development: Mixed Methods Study
Source: J Med Internet Res. 2026 Mar 2;28:e79863. doi: 10.2196/79863 (PMC12954709; doi:10.2196/79863)
Supplement: Multimedia Appendix 1 [file jmir-v28-e79863-s001.pdf]

Search query details for systematic search

A comprehensive search was conducted in PubMed, CINAHL (via EBSCO), and Web of Science, covering publications from March 2014 to March 2024. The search was structured around four key concepts: artificial intelligence, ethics, research, and routinely collected patient data. Relevant MeSH terms, synonyms, and related keywords were identified to develop tailored search strings for each database. All searches were completed on March 6, 2024.

**PubMed**	**Query – last 10 years – 6 March, 2024**	**1895**
1. AI	"Algorithms"[Mesh] OR "artificial intelligence"[Title/Abstract] OR "machine learning"[Title/Abstract] OR "AI"[Title/Abstract] OR "ML"[Title/Abstract] OR "natural language processing"[Title/Abstract] OR "NLP"[Title/Abstract] OR "computational intelligence"[Title/Abstract] OR "Computing Methodologies"[Mesh]	888,429
2. Research	"secondary use"[Title/Abstract] OR "research"[Title/Abstract] OR "studies"[Title/Abstract] OR "study"[Title/Abstract] OR "medical informatics"[Mesh] OR "data sharing"[Title/Abstract]	7,023,232
3. Ethics	"morals*"[Mesh] OR "ethic*"[Title/Abstract] OR "privacy"[Title/Abstract] OR "confidential*"[Title/Abstract] OR "consent"[Title/Abstract] OR "bias"[Title/Abstract] OR "fairness"[Title/Abstract] OR "discriminat*"[Title/Abstract] OR "transparen*"[Title/Abstract] OR "autonomy"[Title/Abstract] OR "responsib*"[Title/Abstract] OR "accountab*"[Title/Abstract] OR "human rights"[Mesh]	786,398
4. Routinely collected health data	"electronic health records"[Mesh] OR "medical records"[Mesh] OR "electronic patient record*"[Title/Abstract] OR "electronic medical record*"[Title/Abstract] OR "electronic health information"[Title/Abstract] OR "routinely collected health data"[Title/Abstract] OR "real-world data"[Title/Abstract] OR "learning health system"[Title/Abstract] OR "EMR"[Title/Abstract] OR "EHR"[Title/Abstract] OR "LHS"[Title/Abstract] OR "Observational data" [Title/Abstract]	89,530

**Web of Science**	**Query – last 10 years – 6 March, 2024**	**1333**

1. AI	1: TS=(“Algorithm*”) OR TS=(“artificial intelligence”) OR TS=(“Machine learning”) OR TS=(“AI”) OR TS=(“ML”) OR TS=(“natural language processing”) OR TS=(“NLP”) OR TS=(“computational intelligence”)	3,079,725
2. Research	2: TS=("secondary use") OR TS=("research") OR TS=("studies") OR TS=("study") OR TS=("medical informatics") OR TS=("data sharing")	22,446,176
3. Ethics	3: TS=("morals") OR TS=("ethic*") OR TS=("privacy") OR TS=("confidential*") OR TS=("consent") OR TS=("bias") OR TS=("fairness") OR TS=("discriminat*") OR TS=("transparen*") OR TS=("autonomy") OR TS=("responsib*") OR TS=("accountab*") OR TS=("human rights")	2,904,808
4. Routinely collected health data	4: TS=("electronic health record*") OR TS=("electronic patient record*") OR TS=("electronic medical record*") OR TS=("electronic health information") OR TS=("routinely collected health data") OR TS=("real world data") OR TS=("learning health system") OR TS=("EHR*") OR TS=("EMR*") OR TS=("LHS") OR TS=("Observational data")	156,583

**CINAHL**	**Query – last 10 years – 6 March, 2024**	**695**
1. AI	(MH "Algorithms") OR (MH "Computing Methodologies+") OR (MH "Natural Language Processing") OR (MH "Machine Learning+") OR (MH "Artificial Intelligence+") OR (TI (artificial intelligence or ai or a.i.) OR AB (artificial intelligence or ai or a.i.) OR TI (machine learning or ml) OR AB (machine learning or ml) OR TI (natural language processing or nlp) OR AB (natural language processing or nlp)) OR (MH "Decision Trees")	194,576
2. Research	(MH "Medical Record Linkage") OR (MH "Secondary Analysis") OR (MH "Research+") OR (MH "Medical Informatics") OR (MH "Electronic Data Interchange+")	1,828,591
3. Ethics	MH "Ethics+" OR MH "Morals+" OR MH "Human Rights+" OR (AB (privacy) OR TI (privacy) OR AB (confidential*) OR TI (confidential*) OR AB (consent) OR TI (consent) OR AB (bias) OR TI (bias) OR AB (fairness) OR TI (fairness) OR AB (discriminat*) OR TI (discriminat*) OR AB (transparen*) OR TI (transperen*) OR AB (autonomy) OR TI	250,091

	(autonomy) OR AB (responsib*) OR TI (responsb*) OR AB (accountab*) OR TI (accountab*))	
4. Routinely collected health data	(MH "Medical Records+") OR (MH "Routinely Collected Health Data") OR (MH "Learning Health System") OR TI (electronic health records) OR AB (electronic health records) OR TI (electronic medical records) or AB (electronic medical records) OR TI (emr) OR AB (emr) OR TI (EHR) OR AB (EHR) OR TI (learning health system) OR TI (LHS) OR AB (learning health system) OR AB (LHS) OR TI (observational data) OR AB (observational data)	80,153
