# Supplementary material for: The Ethics of Leveraging Routinely Collected Patient Data for AI Development: Mixed Methods Study
Source: J Med Internet Res. 2026 Mar 2;28:e79863. doi: 10.2196/79863 (PMC12954709; doi:10.2196/79863)
Supplement: Multimedia Appendix 2 [file jmir-v28-e79863-s002.pdf]

Verslag workshop

Aanpak Begeleidingsethiek

Detecteren van medicatie-gerelateerde nierschade met behulp van routinematig verzamelde zorgdata

Amsterdam UMC

NWO

Amsterdam UMC

ECP
Platform voor de
InformatieSamenleving

Voorwoord

De toenemende digitalisering brengt ingrijpende veranderingen met zich mee, in allerlei sectoren. Nieuwe mogelijkheden voor communicatie, monitoring en analyse roepen vragen op. Wat is de positie van de mens in deze omgeving, hoe staat het met data, met privacy, wat doen algoritmes en wat willen gebruikers eigenlijk? Begeleidingsethiek is ethiek die zich specifiek met dit soort vragen bezighoudt. Hij is gebaseerd op techniekfilosofie, die uitgaat van eeuwenoude verwevenheid tussen mens en technologie.

De Aanpak Begeleidingsethiek is een concrete aanpak waarin betrokkenen met elkaar in dialoog gaan over de effecten van de nieuwe technologie én de waarden die daarbij in het geding komen. Dat zijn vaak waarden gelieerd aan autonomie van de gebruiker, efficiëntie van het proces, transparantie van het algoritme, privacy, et cetera.

Tijdens de sessie komen verschillende stakeholders als gebruikers, ontwikkelaars, beleidsvormers en beslissers met elkaar in gesprek. Na de dialoog hebben de deelnemers ethische handelingsopties gegenereerd, waarvan verschillende vaak direct opgepakt kunnen worden. Er is gezamenlijk gekeken welke waarden we in het digitale domein belangrijk vinden en hoe we die willen verankeren en borgen in digitale processen en handelingen. Dit alles om ook bij verdere digitalisering op het vertrouwen van de samenleving en de participanten kunnen blijven rekenen. Dit is niet in één stap te realiseren, het is een continu proces waarin deze workshop een schakel is.

Workshop aanpak begeleidingsethiek

Initiatiefnemers: Marieke Bak, Ronald Cornet, Joanna Klopotowska en Menno Maris (Amsterdam UMC)
Moderatoren: Pieter van Kuilenburg, Daniël Tijink (ECP)

Op 29 november 2023 organiseerde ECP op initiatief van het team van werkpakket 1 (WP1) van het LEAPfROG-project (Amsterdam UMC) een sessie begeleidingsethiek over de casus 'Detecteren van medicatie-gerelateerde nierschade met behulp van routinematig verzamelde zorgdata'. Aan de workshop namen 18 personen (zie bijlage voor specificatie) deel onder leiding van Pieter van Kuilenburg en Daniël Tijink (ECP).

De doelstelling is tweeledig:

▷ Leren tijdens de workshop

- Welke effecten, waarden en actoren worden genoemd
- Welke handelingsopties zijn er om de toepassing te verbeteren

▷ Leren over de Aanpak Begeleidingsethiek

- Hoe kan de organisatie deze methodiek gebruiken rondom ethische kwesties en technologie-implementaties

Het verslag bevat de volgende elementen:

▷ Toelichting op de Aanpak Begeleidingsethiek
▷ Weerslag van de workshop

Aanpak begeleidingsethiek

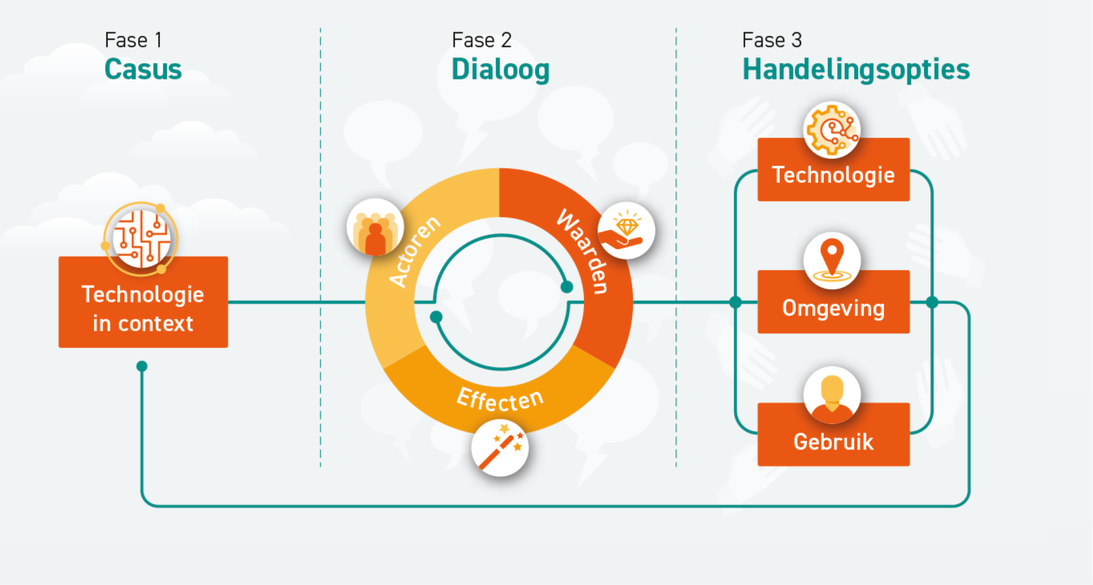

De aanpak bevat de volgende fasen:

Fase 0 Introductie

Introductie over de doelstelling en een toelichting op het model en het gedachtegoed

Fase 1 Toelichting

Hoe ziet de technologie eruit en in welke context wordt deze toegepast

Fase 2 Dialoog

▷Een korte ronde waarin de deelnemers aan de workshop de betrokken actoren benoemen
▷Brainwrite waar deelnemers mogelijke effecten benoemen en bespreken
▷ Benoemen van waarden die een rol spelen bij die effecten

Fase 3 Handelingsopties

In subgroepjes gaan de deelnemers op zoek naar handelingsopties vanuit de technologie, de omgeving en het individu.

Fase 1

Technologie en context

Deze sessie wordt georganiseerd in het kader van het door NWO gefinancierde wetenschappelijk project LEAPfROG. LEAPfROG staat voor **LE**veraging real-world d**A**ta to optimize **P**harmacothe**R**apy outcomes in multim**O**rbid patients by using machine learning and knowled**G**e representation methods.

Het doel van het LEAPfROG-project is om met behulp van routinematig verzamelde zorgdata en innovatieve technologie tot verbeterde inzichten te komen over welke geneesmiddelen veilig en effectief zijn voor patiënten met meerdere chronische aandoeningen (multimorbiditeit). Patiënten met chronische nierschade hebben vaak ook andere chronische aandoeningen, zoals diabetes, hartfalen en hypertensie. Hierdoor gebruiken patiënten met chronische nierschade vaak meerdere geneesmiddelen tegelijkertijd (polyfarmacie). Dit terwijl er weinig bekend is over de veiligheid en effectiviteit van dergelijke complexe geneesmiddelencombinaties. Dit komt doordat patiënten met multimorbiditeit zoals patiënten met chronische nierschade vaak worden uitgesloten van standaard geneesmiddelonderzoek, zoals gerandomiseerde studies. Hierdoor is onder andere onze kennis met betrekking tot de veiligheid van geneesmiddelen voor de nieren beperkt. Dit brengt risico's met zich mee voor nierschade, vooral bij patiënten met chronische nierschade, waar de nieren al minder goed werken.

Als aanvulling op gerandomiseerde studies kan onderzoek met routinematig verzamelde zorgdata uit elektronische patiëntendossiers (EPD's) uitkomst bieden voor patiënten met chronische nierschade en andere patiënten met multimorbiditeit. Deze zorgdata worden door zorgverleners tijdens de routinepatiëntenzorg vastgelegd in het EPD systeem van het ziekenhuis of de huisartspraktijk, afhankelijk van waar de patiënt zich bevindt. Door vervolgens in het kader van wetenschappelijk onderzoek deze zorgdata te analyseren met innovatieve technologieën kunnen we inzicht krijgen in hoe het gebruik van complexe geneesmiddelencombinaties in de praktijk uitpakt en zo de behandeling met geneesmiddelen voor patiënten met chronische nierschadee verbeteren. Dit noemen we secundair gebruik van routinematig verzamelde zorgdata. Echter, om betrouwbare inzichten uit routinematig verzamelde zorgdata te halen, moeten deze gegevens wel van voldoende kwaliteit zijn en toegankelijk zijn voor onderzoekers. Helaas is dit momenteel nog onvoldoende het geval.

Het LEAPfROG-team wil in deze sessie gezamenlijk ethische implicaties van secundair gebruik van routinematig verzamelde zorgdata voor onderzoek in kaart brengen, en verkennen welke effecten dit heeft op verschillende belanghebbenden, waaronder patiënten, artsen, onderzoekers en beleidsmakers. Hierbij worden ook de mogelijke consequenties van het niet gebruiken van routinematig verzamelde zorgdata belicht. We willen onderzoeken wat er nodig is om tot betrouwbare inzichten uit routinematig verzamelde zorgdata te komen, met als uiteindelijk doel de gezondheidszorg te verbeteren en betere beslissingen te ondersteunen voor patiënten met multimorbiditeit zoals patiënten met chronische nierschade. De inzichten die we tijdens deze sessie opdoen, zullen worden gebruikt om digitale hulpmiddelen te ontwikkelen die moeten bijdragen aan de toegankelijkheid en verbetering van de kwaliteit van routinematig verzamelde zorgdata.

Fase 2
Dialoog

In deze tweede fase gaan de deelnemers in gesprek over wie er betrokken zijn bij het verzamelen van routinematige zorgdata en het analyseren ervan voor wetenschappelijk onderzoek (secundair gebruik van routinematig verzamelde zorgdata). Ook buigen zij zich over de positieve en negatieve effecten van het secundair gebruik van routinematig verzamelde zorgdata, en benoemen zij belangrijke waarden waar rekening mee gehouden moet worden bij dergelijk gebruik van zorgdata.

Actoren:

Het onderwerp “actoren” behelst de vraag wie er betrokken zijn of geraakt worden door secundair gebruik van routinematig verzamelde zorgdata in het algemeen, en meer specifiek voor het detecteren van medicatiegerelateerde nierschade. De deelnemers aan tafel vertegenwoordigen al een deel van die actoren. Tijdens de worskhop werden er verschillende betrokkenen benoemd. De toelichting is er naderhand door de projectleider van Leapfrog aan toegevoegd, waardoor het beeld van de rol van de betrokkenen is verrijkt, specifiek voor deze case.

Actor	Betrokken bij/Geraakt door secundair gebruik van routinematig verzamelde zorgdata	Toelichting
Patiënten en patiëntvertegenwoordigers, in het bijzonder patiënten met chronische nierschade	Beide	Steeds vaker worden patiënten betrokken. Dit gebeurt met name door middel van participatie van patiëntvertegenwoordigers in bijv. begeleidingscommissies van wetenschappelijk onderzoeken waarin sprake is van secundair gebruik van zorgdata. Ook kunnen patiënten gevraagd worden hun gegevens in het EPD te controleren. Ze worden geraakt in hun gezondheid door (gebrek aan) inzichten uit de routinematig verzamelde zorgdata.
(Huis)artsen	Beide	(Huis)artsen zijn betrokken omdat zij de zorgdata in het EPD invoeren en zo routinematig verzamelde zorgdata genereren. Deze data kunnen vervolgens voor wetenschappelijk onderzoek (secundair gebruik) worden ingezet. Ze worden geraakt in hun beroepsuitoefening door (gebrek aan) inzichten uit de routinematig verzamelde zorgdata en door het besef dat anderen inzage hebben in en gebruik maken van hun data.
Verpleegkundigen	Beide	Verpleegkundigen zijn ook betrokken omdat zij de zorgdata in het EPD invoeren en daarmee routinematig verzamelde zorgdata genereren. Deze data kunnen vervolgens voor wetenschappelijk onderzoek (secundair gebruik) worden ingezet. Ze worden geraakt

		in hun beroepsuitoefening door (gebreken aan) inzichten uit de routinematig verzamelde zorgdata en door het besef dat anderen inzage hebben in en gebruik maken van hun data.
Apothekers	Beide	Apothekers zijn betrokken omdat zij de zorgdata in het EPD invoeren en daarmee routinematig verzamelde zorgdata genereren. Deze data kunnen vervolgens voor wetenschappelijk onderzoek (secundair gebruik) worden ingezet. Ze worden geraakt in hun beroepsuitoefening door (gebrek aan) inzichten uit de routinematig verzamelde zorgdata en door het besef dat anderen inzage hebben in en gebruik maken van hun data.
Onderzoekers	Beide	Onderzoekers zijn betrokken omdat zij de routinematig verzamelde zorgdata analyseren. Onderzoekers worden in hun vermogen om innovatief onderzoek te doen, en deels daardoor ook in hun academische carrière, beïnvloed door (een gebrek aan) routinematig verzamelde zorgdata.
Datamanagers binnen zorginstellingen of zorgpraktijken	Betrokken	Veel zorginstellingen of zorgpraktijken hebben datamanagers in dienst die onderzoekers ondersteunen bij het toegankelijk en begrijpelijk maken van routinematig verzamelde zorggegevens. Deze zorgdata worden opgeslagen in een database welke beheerd en onderhouden wordt door bijv. de afdeling research datamanagement.
Partijen die databases met routinematig verzamelde zorgdata beschikbaar stellen voor onderzoek, bijv. Stizon, Nivel, Academisch Netwerk Huisartsen, ICUdata, Nefrovisie, NICE	Beide	Deze partijen sluiten overeenkomsten met grote groepen, bijv. huisartspraktijken en/of ziekenhuizen en verzamelen in een centrale database hun zorgdata. Ze zijn betrokken omdat ze het mogelijk maken om grote databases met routinematig verzamelde zorgdata te analyseren. Met name voor de toepassing van innovatieve technieken zoals machine learning zijn grote hoeveelheden zorgdata nodig. Ze worden geraakt in hun dienstverlening/bedrijfsvoering door (gebrek aan) inzichten uit de routinematig verzamelde zorgdata in hun databases.
Leveranciers van EPD-systemen, bijv. EPIC, Chipsoft, Nexus, Promedico, PharmaPartners, CGM	Beide	Leveranciers van EPD-systemen zijn betrokken omdat de manier waarop ze hun systemen bouwen en onderhouden van invloed is op de kwaliteit en toegankelijkheid van zorgdata. Ze worden geraakt in hun marktpositie door (gebrek aan) inzichten uit de routinematig verzamelde zorgdata in hun systemen.
Landelijke overheid zoals Ministerie van VWS	Beide	De landelijke overheid is betrokken omdat zij wetsvoorstellen maakt die de kwaliteit en toegankelijkheid van zorgdata kan bevorderen of juist beperken. Ze kan ook ontwikkelingen en initiatieven starten en/of ondersteunen om zorgdata toegankelijk te maken voor onderzoek; bijv. Health-RI of European Health Data Space. De overheid wordt in haar zorgplicht geraakt door (gebrek aan) inzichten uit de routinematig verzamelde zorgdata.
Lokale overheden zoals Gemeente Amsterdam	Beide	Lokale overheden zijn betrokken omdat zij de geldende wet- en regelgeving lokaal moeten uitvoeren. Ze kunnen ook ontwikkelingen en/of initiatieven om zorgdata voor onderzoek toegankelijk te maken in hun gemeente starten en/of ondersteunen; bijv. Health Data Space Amsterdam (HDSA). Lokale overheden worden in hun zorgplicht geraakt door (gebrek aan) inzichten uit de routinematig verzamelde zorgdata.
Bestuurders in de zorg, bijv. Raad van Bestuur in het ziekenhuis	Beide	Bestuurders in de zorg zijn betrokken omdat zij de data infrastructuur binnen hun organisaties geschikt moeten maken voor onderzoek, in overeenstemming met de geldende wet- en regelgeving. Bestuurders in de zorg moeten ook zorgen dat patiënten en zorgverleners erop kunnen vertrouwen dat er zorgvuldig wordt omgegaan met zorgdata en dit vertrouwen niet verliezen. Ze worden in hun bedrijfsvoering en zorgplicht geraakt door (gebrek aan) inzichten uit de routinematig verzamelde zorgdata.
Functionarissen	Betrokken	Functionarissen gegevensbescherming/privacy zijn betrokken omdat

gegevensbescherming/privacy		zij erop moeten toezien dat er binnen hun organisaties zorgvuldig wordt omgegaan met routinematig verzamelde zorgdata.
Medisch Ethische Toetsingscommissies (METC)	Betrokken	METC's zijn betrokken omdat zij beoordelen of een voorstel voor wetenschappelijk onderzoek met zorgdata voldoet aan geldende wet- en regelgeving en ethische kaders. METC's kunnen de desbetreffende onderzoeken goed- of afkeuren.
Zorgverzekeraars	Beide	Zorgverzekeraars verzamelen ook zorgdata die ze voor onderzoek kunnen gebruiken, waardoor ze ook betrokken zijn bij secundair gebruik. Zorgverzekeraars worden in hun bedrijfsvoering en zorgplicht geraakt door (gebrek aan) inzichten uit de routinematig verzamelde zorgdata.
Media en actiegroepen	Geen van beide	Hoewel de media niet direct betrokken zijn of geraakt worden door secundair gebruik van routinematig verzamelde zorgdata, kunnen ze de publieke opinie in zowel negatieve als positieve zin beïnvloeden. Hierdoor kan secundair gebruik stagneren of juist versnellen. Dit geldt ook voor de actiegroepen.
Toezichthouders in de zorg zoals Nederlandse Zorgautoriteit of IGJ	Betrokken	Toezichthouders in de zorg zijn betrokken omdat ze via hun instrumenten en regels secundair gebruik van routinematig verzamelde zorgdata kunnen bevorderen of beperken.
Farmaceutische industrie	Beide	De farmaceutische industrie is betrokken omdat zij via subsidies onderzoek naar geneesmiddelen met routinematig verzamelde zorgdata kan stimuleren. Ze wordt geraakt in haar bedrijfsvoering door (gebrek aan) inzichten uit de routinematig verzamelde zorgdata.
MedTech bedrijven, bijv. Castor, Medscio	Beide	MedTech bedrijven zijn binnen deze context bedrijven die producten en diensten aanbieden met als doel de kwaliteit en toegankelijkheid van zorgdata voor onderzoek te bevorderen. Daarom worden zij gezien als betrokken partij. Ze worden geraakt in hun bedrijfsvoering door (gebrek aan) inzichten uit de routinematig verzamelde zorgdata die mede door hun diensten of oplossingen zijn verkregen.
Onderzoeksfinanciers zoals NWO, ZonMW	Betrokken	Onderzoeksfinanciers zijn betrokken omdat zij voorwaarden stellen aan de beschikbaarheid van onderzoeksgegevens tijdens en na afloop van door hen gefinancierd onderzoek. Dit geldt ook voor routinematig verzamelde zorgdata die voor onderzoek gebruikt worden. Bovendien kunnen ze subsidies verstrekken voor onderzoek om de kwaliteit en toegankelijkheid van zorgdata voor onderzoek te verbeteren.

De vraag aan de deelnemers was om in het vervolg van de sessie ook te proberen vanuit deze perspectieven hun inbreng te geven.

Effecten:

De vraag is hier welke positieve en negatieve effecten secundair gebruik van routinematig verzamelde zorgdata kan hebben. Er werd ook gesproken over de gevolgen van het niet gebruiken van routinematig verzamelde zorgdata voor secundaire doeleinden zoals wetenschappelijk onderzoek. De deelnemers benoemden de volgende positieve en negatieve effecten.

Positieve effecten

- Winst van gezondheid en kwaliteit van leven.
- Bevordert eigen regie v.d. patiënt, want inzichten sluiten beter aan op de situatie van een patiënt met chronische nierschade.
- Meer vertrouwen in arts want minder geneesmiddelbijwerkingen op de nieren.
- Verbeterde beslissingsondersteuning in de spreekkamer, waardoor artsen minder tijd nodig hebben om de juiste medicijnen voor te schrijven aan patiënten met een chronische nierziekte.
- Minder zorgkosten.
- Vergroot maatschappelijke betrokkenheid van patiënten doordat ze hun zorgdata controleren en beschikbaar stellen voor onderzoek.
- Minder druk op mantelzorgers.
- Wetenschappelijke vooruitgang (nieuwe kennis, innovatie), wat positief is voor onderzoekers maar ook een zorginstelling als Amsterdam UMC en Nederland als geheel op de kaart kan zetten als kennisland.
- Inclusiever medisch onderzoek,
- Projecten als LEAPfROG stimuleren initiatieven om tot verbetering in de kwaliteit en toegankelijkheid van routinematig verzamelde zorgdata te komen.

Negatieve effecten

- Bias in- en lage kwaliteit van de routinematig verzamelde zorgdata leiden tot onbetrouwbare inzichten over de veiligheid van geneesmiddelen voor de nieren.
- Digistarters worden mogelijk benadeeld (geldt voor zowel zorgprofessionals als patiënten) omdat zorgdata van digistarters mogelijk van slechtere kwaliteit zijn.
- Zelfbeschikking van de patiënten over zorgdata komt in het geding.
- Te veel hoop geven aan patiënten over de vermijdbaarheid van schade aan de nieren door geneesmiddelen.
- Te veel vertrouwen in inzichten uit data (kwantitatief mensbeeld).
- Overvragen van de patiënt en zorgverleners om kwaliteit van zorgdata te bevorderen.
- Artsen moeten gegevens beter registreren waardoor meer werk.
- Werktevredenheid van zorgverleners vermindert door minder autonomie bij het vastleggen van zorgdata in EPD
- Rol van behandelaar verandert door gebruik van deze data op een manier die hij-zij niet prettig vindt
- Privacy van de patiënten komt in het geding.

- Bevindingen uit onderzoek bereiken sneller de zorgpraktijk.
- Economische groei ten behoeve van actoren zoals partijen die databases beschikbaar stellen voor onderzoekers, EPD leveranciers en MedTech bedrijven. Zorginstellingen kopen EPD systemen; onderzoekers maken gebruik van zorgdata en profiteren van de oplossingen om de kwaliteit en toegankelijkheid van gezondheidszorggegevens te verbeteren.

- Misbruik van gegevens, bijv. onbedoeld gebruik door private partijen en zorgverzekeraars.
- Geld gaat naar de verkeerde "oplossing", misschien is er bijv. meer geld nodig voor verzorging o.p.v. onderzoek met zorgdata
- Reputatie- en ook financiële schade voor actoren (bijv. Amsterdam UMC, partijen die databases beschikbaar stellen voor onderzoek, EHR-leveranciers, MedTech) als inzichten uit zorgdata onbetrouwbaar zijn omdat kwaliteit en toegankelijkheid van data of oplossingen onvoldoende zijn.
- Nederland gaat wetenschappelijk achteruit omdat in vergelijking met andere landen de infrastructuur voor secundair gebruik van zorgdata onvoldoende is om betrouwbare inzichten te verkrijgen

Waarden:

De relevante waarden zijn door de co-moderator geïnventariseerd terwijl de deelnemers bezig waren met het benoemen van effecten. Achter veel effecten gaan waarden schuil. Die lijst is gedeeld met de deelnemers en door hen besproken en aangevuld. Dat leverde de volgende lijst met waarden op:

**Waarden**	
Gezondheid	Veiligheid (medisch)
Kwaliteit van leven	Veiligheid (data)
Betrouwbaarheid	Privacy
Autonomie	Kwaliteit van zorg
Vertrouwen	Wetenschappelijk statuur van Nederland
Betrokkenheid	Rechtvaardigheid
Kennis/innovatie	Effectiviteit
Werkplezier	Solidariteit
Efficiëntie	Toegankelijkheid

Tijdens een korte inventarisatie werden **kwaliteit van leven, autonomie, vertrouwen, privacy, kwaliteit van zorg en solidariteit** door deelnemers als belangrijkste waarden genoemd binnen de context van deze sessie.

Fase 3

Handelingsopties

In het proces van het opstellen van de handelingsopties zijn de belangrijkste waarden uit de vorige fase meegenomen. De deelnemers kregen een uitleg over de verschillende categorieën binnen de handelingsopties. Het kan bijvoorbeeld gaan over het ontwerp van de technologie, het aanpassen van de omgeving waarbinnen de toepassing functioneert en het gedrag van mensen. De deelnemers werden in drie werkgroepen verdeeld en gingen ieder aan de slag met een van deze drie gespreksonderwerpen. Dat leidde tot handelingsopties per categorie.

Ontwerpen van technologie: ethics by design

Hoe technologie zo te ontwerpen dat zij beter aansluit bij de gekozen waarden.

- Bouw in EPD systemen functionaliteit in waarmee patiënten zelf toestemming kunnen geven of intrekken voor het gebruik van hun zorgdata (*autonomie, privacy, vertrouwen, solidariteit*).
- EPD systemen worden zo gebouwd dat ze zorgdata registratie door zorgverleners sterk in kwaliteit bevorderen en tegelijkertijd intuïtief en gemakkelijk maken (*kwaliteit van zorg*)
- EPD systemen worden zo gebouwd dat ze betrouwbare, veilige en volledige uitwisseling van zorgdata tussen verschillende system mogelijk maken (*kwaliteit van zorg, kwaliteit van leven, vertrouwen, solidariteit*)
- Creëer oplossingen (bijv. een dashboard) waarmee het combineren van zorgdata uit verschillende EPD systemen inzichtelijk is voor de patiënt. Daarbij moet helder worden welke zorgdata worden verzameld, door wie en voor welk doel, en moet de patiënt daar toegang toe hebben (*autonomie, vertrouwen, solidariteit*).
- Ontwikkel oplossingen om kwaliteitsissues en/of vertekening (bias) in routinematig verzamelde zorgdata (zoals data uit EPD systemen) inzichtelijk te maken voor de eindgebruikers van deze data en waar mogelijk deze issues te verhelpen (*vertrouwen, kwaliteit van zorg, kwaliteit van leven*)
- EPD systemen en databases met EPD data worden zo gebouwd dat risico's op datalekken worden geminimaliseerd en de zorgdata voor onderzoek ontdaan wordt van tot de individuele patiënt herleidbare gegevens d.m.v. pseudonimisatie of anonimisatie (*vertrouwen, privacy, kwaliteit van zorg*).

- Creëer digitale zorgdata omgevingen (bijv. virtual research clouds) die voldoen aan wet- en regelgeving en oneigenlijk of onjuist gebruik van de zorgdata minimaliseren (*vertrouwen, privacy, kwaliteit van zorg*).
- Creëer een mogelijkheid voor de patiënten om kwaliteit van eigen zorgdata in het EPD te controleren en bevindingen te communiceren naar zorgverleners (*autonomie, vertrouwen, solidariteit*).

Omgeving: inrichten en afspraken maken; ethics in context

Hoe de context waarin technologie wordt gebruikt (fysiek, sociaal, beleid/ juridisch) aan te passen om de gekozen waarden beter te weerspiegelen.

- Maak een afsprakenstelsel voor pseudonimisatie/anonimisatie van zorgdata voor onderzoek. Liefst nationaal, in ieder geval regionaal (*vertrouwen, privacy, kwaliteit van zorg*).
- Stel kwaliteitsnormen op voor de digitale zorgdata omgevingen waarin met zorgdata gewerkt wordt. Maak afspraken met eindegebruikers en leveranciers van deze omgevingen over hoe deze normen kunnen worden "afgedwongen" (*kwaliteit van zorg, vertrouwen, privacy*).
- Bevorder digitale vaardigheden van patiënten en zorgverleners (*autonomie, kwaliteit van zorg, kwaliteit van leven, solidariteit*).
- Ondersteun en beloon zorgverleners bij goed gebruik van EPD systeem (*kwaliteit van zorg*).
- Spreek met alle stakeholders af *welke* data worden vastgelegd in het EPD systeem en *waar* en *hoe* dit gebeurt. Toon daarmee leiderschap in het creëren van eenheid van taal (*solidariteit, vertrouwen, kwaliteit van zorg, kwaliteit van leven*).
- Leg metadata (data over de data) vast en spreek ook daarbij af: welke metadata wordt door wie gebruikt? (*vertrouwen, solidariteit*)
- Ontwikkel een visie op het 'waarom' van secundair gebruik van routinematig verzamelde zorgdata, bijvoorbeeld via een consensus sessie met patiënten, zorgverleners, onderzoekers, , data-managers, en functionarissen gegevensbescherming (*vertrouwen, solidariteit, kwaliteit van zorg, kwaliteit van leven, privacy*).
- Organiseer voorlichting aan patiënten en zorgverleners (bijv. een data-café) in het kader van verwachtingsmanagement (korte/lange-termijn impact van secundair gebruik van routinematig verzamelde zorgdata voor individu/collectief) en grote (o.a. epidemiologische) vraagstukken (wat is kennis en wat kan geleerd worden uit routinematig verzamelde zorgdata; wat levert het "inzicht"

op voor wie?). Incl. reflectie over welke handelingsopties er zijn en voor wie (*vertrouwen, solidariteit, kwaliteit van zorg, kwaliteit van leven*).

- Bouw controlestappen en toezicht in het proces van secundair gebruik van routinematig verzamelde zorgdata (*vertrouwen, privacy, kwaliteit van zorg*).
- Ga uit van het principe van ‘evoluerende technologie’; blijf de impact op de zorg en eindgebruikers van secundair gebruik van routinematig verzamelde zorgdata evalueren en verbeteren waar nodig (*vertrouwen, solidariteit, privacy, kwaliteit van zorg*).
- Creëer bij voorkeur één identificatienummer waardoor je een patiënt kunt volgen in de levensloop of reis langs alle zorgverleners (zoals in Denemarken), (*kwaliteit van zorg, kwaliteit van leven*).
- Harmoniseer uitvoering van wet- en regelgeving m.b.t. secundair gebruik van routinematig verzamelde zorgdata. Nu is er te veel interpretatie door individuen waardoor secundair gebruik belemmerd wordt (*solidariteit, kwaliteit van zorg, kwaliteit van leven*).

Gebruiker: bewust worden en gedrag aanpassen: ethics by user

Wat kan de mens doen ten aanzien van het gebruik van technologie zodat het gebruik ervan beter aansluit bij de gekozen waarden.

- Organiseer als data onderzoeker een bewustwordingscampagne bijv. in samenwerking met wetenschappelijke verenigingen en patiëntenverenigingen. Dit om het belang van secundair gebruik van routinematig verzamelde zorgdata toe te lichten aan patiënten en zorgverleners (*vertrouwen, solidariteit, kwaliteit van zorg, kwaliteit van leven*).
- Praat met elkaar als zorgverleners over het juist gebruik van het EPD-systeem, en spreek elkaar aan bij onjuist gebruik die de kwaliteit van zorgdata verminderd (*solidariteit, kwaliteit van zorg)*.
- Ga als functionaris gegevensbescherming/privacy officer in dialoog met onderzoekers en zorgverleners i.p.v. alleen te praten met RvB over de voor- en nadelen van (niet) gebruik van routinematige verzamelde zorgdata voor onderzoek. Dit om wederzijds begrip te bevorderen en zo tot betere afspraken rond zorgdatabeheer en -gebruik te komen (*vertrouwen, solidariteit, kwaliteit van zorg*).
- Communiceer als onderzoeker breed de tussen- en eindresultaten van projecten waarin routinematig verzamelde zorgdata zijn gebruikt (zoals LEAPfROG). Wat ging goed, en wat kan beter? (*vertrouwen, kwaliteit van zorg, kwaliteit van leven*)
- Betrek als onderzoekers de eindgebruikers op wie de verwachte inzichten uit routinematig verzamelde zorgdata betrekking hebben

zoveel mogelijk vanaf het begin bij het project (*vertrouwen, solidariteit, kwaliteit van zorg, kwaliteit van leven*).

- Integreer de inzichten uit onderzoek en ontwikkelde oplossingen in bestaande workflows en/of bestaande software zoals EPD systemen of patiëntenportalen (*kwaliteit van zorg, autonomie*).

Terugblik en afronding

Aan het einde van de workshop wordt teruggekeken en besproken wat de Aanpak Begeleidingsethiek concreet heeft opgeleverd. De deelnemers geven aan dat het veel nieuwe inzichten oplevert en dat er in korte tijd veel wordt gerealiseerd. Daarnaast wordt de diversiteiten van de groep van deelnemers als meerwaarde ervaren. De combinatie van een duidelijke casus, enthousiaste deelnemers, en een aanpak die zorgt voor duidelijke uitkomsten in een korte tijd was dus een succes.

De deelnemers

1. **Joanna Klopotowska** senior onderzoeker en projectleider LEAPfROG, Amsterdam UMC
2. **Ronald Cornet** senior onderzoeker en leider werkpakket 1 binnen LEAPfROG, Amsterdam UMC
3. **Marieke Bak** medisch ethicus en senior onderzoeker, Amsterdam UMC
4. **Menno Maris** PhD-kandidaat medische ethiek, Amsterdam UMC
5. **Joris Lieverse** PhD-kandidaat LEAPfROG, Amsterdam UMC
6. **Azam Nurmohamed** internist-nefroloog en Chief Medical Information Officer (CMIO), Amsterdam UMC
7. **Nicolette de Keizer** hoogleraar medische informatiekunde, Amsterdam UMC
8. **David de Koning** datamanagement, Amsterdam UMC
9. **Martin den Heijer** hoogleraar endocrinologie en Chief Scientific Information Officer (CSIO), Amsterdam UMC
10. **Lies van Gennip** Kwartiermaker Health Data Space Amsterdam (HDSA), Amsterdam Economic Board
11. **Wanda Konijn** patiëntvertegenwoordiger, Nierpatiënten Vereniging Nederland (NVN):
12. **Clary Idzinga** ervaringsdeskundige chronische nierschade, NVN
13. **Bart Kranenburg** ervaringsdeskundige chronische nierschade, NVN
14. **Lucas Fleuren** medeoprichter Medscio
15. **Tristan Duchenne** Information Security Officer, Sigra
16. **Romy Vos** PhD-kandidaat LEAPfROG, Vrije Universiteit
17. **Roos de Jong** junior manager digital ethics, Deloitte

Moderatoren:
Pieter van Kuilenburg, Daniël Tijink (ECP)

aanpak begeleidings ethiek

Verslag workshop
Aanpak Begeleidingsethiek

Detectie van medicatie-gerelateerde nierschade met behulp van routinematig verzamelde zorgdata

Meer informatie over de Aanpak Begeleidingsethiek, waaronder dit verslag, vindt u op
www.begeleidingsethiek.nl

ECP
Platform voor de InformatieSamenleving
