# Supplementary material for: The Ethics of Leveraging Routinely Collected Patient Data for AI Development: Mixed Methods Study
Source: J Med Internet Res. 2026 Mar 2;28:e79863. doi: 10.2196/79863 (PMC12954709; doi:10.2196/79863)
Supplement: Multimedia Appendix 3 [file jmir-v28-e79863-s003.pdf]

# LEAPfROG project

Workshop begeleidingsethiek

10 Oktober 2024

# Inhoud

Inleiding ... 3
Hoe deze workshop was opgezet ... 4
Fase 1 ... 5
**1.1 Achtergrond** ... 5
**1.2 Vragenronde: verkenning en verdieping** ... 6
Fase 2 ... 8
**2.2 Actoren** ... 8
**2.2 Mogelijke Effecten** ... 9
Fase 3 ... 12
**3.1 Handelingsopties** ... 12
Reflectie op de Workshop door Onderzoekers van het LEAPfROG-project ... 17
Bijlage 1. Deelnemers ... 18

## Inleiding

Op 10 oktober 2024 vond een workshop begeleidingsethiek plaats in het kader van het LEAPfROG-project ("LEveraging real-world dAta to optimize PharmacotheRapy outcomes in multimOrbid patients by using machine learning and knowledGe representation methods"). LEAPfROG is een wetenschappelijk onderzoek naar de meerwaarde van machine learning en big data voor het optimaliseren van farmacotherapie-uitkomsten bij patiënten met multimorbiditeit. Het doel van de workshop was om met een diverse groep belanghebbenden in gesprek te gaan over de ethische overwegingen die kunnen ontstaan bij het gebruik van een door LEAPfROG te ontwikkelen causaal machine learning-model voor de achterafdiagnose van door medicijnen veroorzaakte acute nierfalen bij patiënten met chronische nierziekte. Binnen LEAPfROG is gekozen voor deze use-case als voorbeeld van een farmacotherapie-uitkomst bij patiënten met multimorbiditeit. Met achteraf wordt bedoeld dat de diagnose "medicatie-veroorzaakt" wordt gesteld nadat acute nierfalen heeft plaatsgevonden. Het vaststellen van dergelijke achterafdiagnoses is essentieel om betere en uitgebreidere kennis en data te verkrijgen over acute nierfalen veroorzaakt door medicijnen bij patiënten met chronische nierziekte. Dit maakt het mogelijk om bij toekomstige patiënten de kans op acute nierfalen door medicijnen te voorspellen en zo deze schadelijke gebeurtenissen te voorkomen met behulp van prognostische modellen.

De workshop bouwde voort op een eerdere workshop begeleidingsethiek, georganiseerd binnen het LEAPfROG-project, die plaatsvond op 30 november 2023. Tijdens die eerdere bijeenkomst stonden ethische overwegingen bij het hergebruik van routinematig verzamelde zorgdata uit het elektronisch patiëntendossier centraal. De workshop werd geleid door vertegenwoordigers van het Platform voor de InformatieSamenleving (ECP) en was gebaseerd op de door ECP ontwikkelde methodiek Aanpak Begeleidingsethiek. Deze methode streeft naar technologische ontwikkelingen die worden gestuurd door ethische overwegingen en zoveel mogelijk aansluiten bij de waarden van betrokken belanghebbenden.

In de vervolgworkshop, waarvan we de resultaten in dit verslag presenteren, is de methode Aanpak Begeleidingsethiek opnieuw als basis genomen. Dit keer werd de workshop geleid door LEAPfROG-projectleider Joanna Klopotowska en Menno Maris, onderzoeker van de afdeling Ethiek, Recht en Humaniora van het Amsterdam UMC. Dertien deelnemers namen deel aan de workshop.

De deelnemersgroep heeft, in gesprek met LEAPfROG-onderzoekers, uitgebreid stilgestaan bij de context waarin het causale machine learning-model mogelijk zal worden ingezet. Er werd gesproken over de werking van het beoogde model en de mogelijke positieve en negatieve effecten die de ontwikkeling en het gebruik ervan met zich mee kunnen brengen. Aan het einde van de workshop is een eerste stap gezet in het bedenken van handelingsopties om de positieve impact van het model zoveel mogelijk te waarborgen en de negatieve effecten te beperken. Daarnaast zijn manieren overwogen om deze inzichten te integreren in de verdere ontwikkeling van het causale machine learning-model binnen het LEAPfROG-project.

De uitkomsten van beide workshops worden gecombineerd met een literatuurstudie. Samen vormen deze resultaten de basis voor een wetenschappelijk paper waarin de inzichten uit de sessies en de literatuur worden geïntegreerd. Het verslag van de eerste workshop van 30 november 2023 is beschikbaar via https://www.pharmacoinformaticslab.nl/leapfrog/partners/.

# Hoe deze workshop was opgezet

**Fase 1.** ***Technologie in Context; Verkenning en Verdieping***

- Een beschrijving van het causale LEAPfROG machine learning-model voor achterafdiagnose van door medicatie veroorzaakte acute nierinsufficiëntie bij patiënten met chronische nierziekte, evenals de beoogde zorgcontext waarin dit model mogelijk zal worden toegepast.
- Presentatie van de belangrijkste resultaten uit de eerste workshop.
- Verdiepende vragenronde voor de deelnemers.

**Fase 2.** ***Actoren en Effecten***[1]

- *2.1 Actoren*
  Een overzicht opstellen van actoren van wie het perspectief in overweging moet worden genomen.
- *2.2 Effecten*
  Het in kaart brengen van zowel de mogelijke positieve als negatieve effecten van het gebruik van de technologie in de context.

**Fase 3.** ***Handelingsopties***

- *Op drie niveaus:*
  - Technologie “ethics by design” – (ontwerp van het model, bijvoorbeeld in termen van werking of presentatie).
  - *Omgeving “ethics in context” – (bijv. richtlijnen of regelgeving, maar ook inbedding van het causale model in bestaande systemen).*
  - *Gebruiker “ethics by user” – (bijv. gebruiksnormen of educatie).*

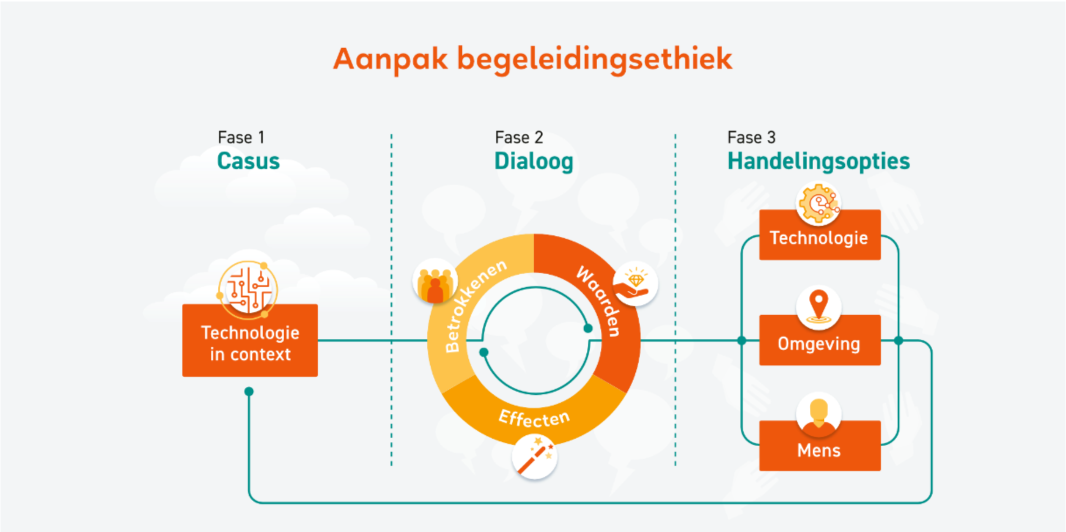


Fig 1. Aanpak Begeleidingsethiek - https://begeleidingsethiek.nl/

[1] Vanwege tijdsbeperking is de stap ‘waarden’ uit fase 2 niet expliciet als losstaand onderdeel opgenomen. Ons ontwerp wijkt daardoor licht af van de methode Aanpak Begeleidingsethiek.

# Fase 1

## 1.1 Achtergrond

Om in gesprek te kunnen gaan over de ethische aspecten van het gebruik van een technologie en hoe hiermee om te gaan, is het essentieel om de technologie en de context waarin deze wordt ontwikkeld en gebruikt goed te begrijpen.

Ter voorbereiding op de workshop, ontvingen de deelnemers onderstaande casusbeschrijving over het causaal machine learning model voor achterafdiagnose van acuut nierfalen door medicatie bij patiënten met chronische nierziekte. Het doel van LEAPfROG is om met behulp van innovatieve technologie tot verbeterde inzichten te komen over welke geneesmiddelen veilig en effectief zijn voor patiënten met meerdere chronische aandoeningen. Onderdeel hiervan is ontwikkelen van een causaal machine learning model dat artsen helpt vast te stellen of acuut nierfalen door medicijnen is veroorzaakt in een patiënt met chronische nierziekte, ook wel een "achterafdiagnose" genoemd. Dit model helpt bij het onderscheiden van door medicijnen veroorzaakte nierfalen van andere oorzaken van nierfalen en bouwt zo een dataset op van patiënten met en zonder door medicatie-veroorzaakt acuut nierfalen.

Fig. 2 Casusbeschrijving

***Casusbeschrijving - "Causaal machine learning-model voor achterafdiagnose van acuut nierfalen door medicatie bij patiënten met chronische nierziekte"***

***Achtergrond***

*Deze workshop begeleidingsethiek wordt georganiseerd in het kader van het wetenschappelijke project LEAPfROG. LEAPfROG staat voor LEveraging real-world dAta to optimize PharmacotheRapy outcomes in multimOrbid patients by using machine learning and knowledGe representation methods.*

*Het doel van het LEAPfROG-project is om met behulp van innovatieve technologie tot verbeterde inzichten te komen over welke geneesmiddelen veilig en effectief zijn voor patiënten met meerdere chronische aandoeningen. Patiënten met chronische nierziekte hebben vaak andere chronische aandoeningen, zoals diabetes, hartfalen en hypertensie. Hierdoor gebruiken patiënten met chronische nierziekte vaak meerdere geneesmiddelen tegelijkertijd. Tegelijkertijd is er weinig bekend over de veiligheid en effectiviteit van dergelijke complexe geneesmiddelencombinaties. Dit komt doordat patiënten met chronische nierziekte vaak worden uitgesloten van standaard geneesmiddelonderzoek, zoals gerandomiseerde studies. Hierdoor is onder andere onze kennis met betrekking tot de veiligheid van geneesmiddelen voor de nieren beperkt. Dit brengt risico's met zich mee voor de nieren, vooral bij patiënten met chronische nierziekte bij wie de nieren al minder goed werken.*

*Om dit probleem aan te pakken, richt LEAPfROG zich op het gebruik van gegevens uit elektronische patiëntendossiers (EPD's). Deze bevatten waardevolle informatie over patiënten met chronische nierziekte en andere aandoeningen. Met behulp van deze data ontwikkelen we een causaal machine learning-model dat artsen helpt vast te stellen of acuut nierfalen door medicijnen is veroorzaakt. Dit noemen we een "achterafdiagnose", omdat het model wordt gebruikt nadat acuut nierfalen al is opgetreden. "Causaal" betekent dat het model helpt om de oorzaak-gevolgrelatie te voorspellen. Ons doel is om een dataset te creëren van patiënten met en zonder acuut nierfalen door medicijnen. Een dergelijke dataset bestaat momenteel niet, maar is essentieel voor het ontwikkelen van*

*toekomstige machine learning-modellen die kunnen voorspellen welke patiënten risico lopen op acuut nierfalen door medicijnen. Op die manier kunnen deze schadelijke gebeurtenissen worden voorkomen.*

***Het doel van de workshop***
*Tijdens de ethiekbegeleidingsworkshop willen we graag met u in gesprek over de mogelijke ethische kwesties die kunnen ontstaan wanneer ons causale machine learning-model voor de achterafdiagnose van acuut nierfalen door medicijnen bij patiënten met chronische nierziekte in gebruik wordt genomen. We horen graag de perspectieven van verschillende belanghebbenden, zoals patiënten, artsen, onderzoekers en beleidsmakers. Nadat we de ethische kwesties hebben verkend, willen we samen met u nadenken over mogelijke oplossingen, die we vervolgens proberen te integreren in de verdere ontwikkeling van ons causale machine learning-model binnen het LEAPfROG-project.*

## 1.2 Vragenronde: verkenning en verdieping

**De workshop werd geopend met een presentatie van projectleider Joanna Klopotowska, waarin het beoogde causale model en de bredere inbedding ervan binnen het LEAPfROG-project werden toegelicht. Na afloop van de presentatie kregen de deelnemers de gelegenheid om verdiepende vragen te stellen, eventuele onduidelijkheden te verhelderen en in gesprek met de onderzoekers de casus verder uit te diepen. Hieronder volgt een overzicht van de thema's die tijdens dit verdiepende gesprek het meest uitvoerig zijn besproken.**

***1.2.1 Registratielast.*** Om een betrouwbaar causaal machine learning-model voor medicatiebijwerkingen te ontwikkelen dat van toegevoegde waarde kan zijn binnen de huidige klinische praktijk, zou je idealiter gebruik willen maken van betrouwbare EPD-data, met name van registraties van medicatiebijwerkingen. Volgens de LEAPfROG-onderzoekers zijn dergelijke data nauwelijks beschikbaar. Een huisarts merkte op dat er tegenwoordig sterk wordt aangestuurd op zorg rondom patiënten met polyfarmacie, maar benadrukte dat de gedetailleerde registratie van bijwerkingen buitengewoon tijdrovend kan zijn en tot wel 30 minuten in beslag kan nemen. Het verder verhogen van deze registratielast ten behoeve van onderzoek zou in de praktijk vrijwel onwerkbaar zijn. Er zal dus moeten worden nagedacht over manieren waarop zorgverleners kunnen worden ondersteund in het verhogen van de datakwaliteit rondom medicatiebijwerkingen, zonder dat dit de administratielast van zorgverleners verhoogt.

***1.2.2 Mogelijk confronterend voor artsen.*** Deelnemers gaven aan dat het gebruik van het causale machine learning-model mogelijk een gevoel van kwetsbaarheid bij zorgverleners kan veroorzaken ten aanzien van hun positie in het zorgproces. Hoewel het model als diagnosehulpmiddel voor artsen wordt gepresenteerd, kan het ook confronterend zijn wanneer artsen geconfronteerd worden met hun eerdere medicatiekeuzes die, gebaseerd op eerdere beslissingen, negatief zijn uitgepakt en mogelijk acuut nierfalen hebben veroorzaakt. Dit kan artsen bovendien kwetsbaar maken ten aanzien van patiënten en collega's, vooral wanneer medicatiefouten herleidbaar zijn naar de keuzes van een individuele arts.

***1.2.3 Mogelijk leereffect bij inzet causaal machine learning-model.*** Deelnemers zagen echter ook veel potentieel in het beoogde model voor het verwerken en combineren van grote hoeveelheden medische data in patiëntendossiers (bijv. medicatiegebruik, laboratoriumresultaten en resultaten van andere diagnostische tests) en medische domeinkennis (bijv. kennis uit richtlijnen, medische naslagwerken en wetenschappelijke publicaties) en het presenteren ervan in een gestructureerde en toegankelijke vorm voor zorgverleners. Door opmerkelijke patronen te signaleren, zou het model kunnen dienen als een startpunt voor het bevestigen van vermoedens of het aanzetten tot verder diagnostisch onderzoek om de oorzaak van acuut nierfalen te kunnen vaststellen. Wanneer

het model op deze manier wordt ingezet, zou het zelfs een leereffect kunnen veroorzaken bij zorgverleners, wat hun begrip van medicatiebijwerkingen in de toekomst zou kunnen verbeteren. Dit zou uiteindelijk ook de kwaliteit van de zorg ten goede komen. Aanwezigen benadrukten dat hoewel het model nuttige suggesties kan bieden, het belangrijk is om de resultaten in combinatie met klinische expertise te beoordelen.

***1.2.4 Ondersteunende rol in shared decision-making.*** Zowel patiëntvertegenwoordigers als zorgverleners merkten op dat patiënten over het algemeen goed geïnformeerd willen worden, zelfs wanneer het om ongunstig nieuws gaat. Wanneer er transparant naar de patiënt over het gebruik van het model wordt gecommuniceerd, waarbij de uitkomsten en de overwegingen van de arts worden toegelicht, zou het gebruik van het model gezamenlijke besluitvorming zelfs kunnen ondersteunen en, vergelijkbaar met het leereffect bij artsen, het begrip van patiënten voor de redenering achter hun behandeling kunnen vergroten, vertrouwen bevorderen en mogelijk de naleving van medische aanbevelingen verbeteren.

***1.2.5 De presentatie van modeluitkomsten.*** Deelnemers maakten zich zorgen over hoe de probabilistische uitkomsten (bijv. een 60% waarschijnlijkheid van een causaal verband tussen medicatie en acuut nierfalen) naar zorgverleners en patiënten worden gecommuniceerd. De perceptie van percentages en de presentatie van mogelijke oorzaken moet zorgvuldig worden overwogen om verkeerde interpretaties te voorkomen. Daarnaast werd gediscussieerd over het beste moment om de voorspelling te geven: bijv. tijdens de opname of bij het ontslag uit het ziekenhuis. Bij het ontslag zullen de eindgebruikers nog nauwelijks handelingsopties hebben buiten de registratie van de bijwerking, maar kan het model wel meer data benutten. Tijdens de opname, hoewel de data over de opname nog niet compleet zijn, kunnen de eindgebruikers in sommige gevallen nog wel handelingsopties hebben, zelfs als acuut nierfalen zich al heeft voorgedaan.

Bijvoorbeeld bij een patiënt die voor een infectie behandeld wordt met een voor de nieren risicovol antibioticum en acuut nierfalen ontwikkelt terwijl er een nierveilig alternatief bestaat, zal een voorspelling van het model van klinische meerwaarde zijn, zeker als de arts nog niet heeft herkend dat acuut nierfalen mogelijk medicatiegerelateerd is. De arts kan besluiten om te switchen. Een voorspelling bij een patiënt met sepsis waarbij het niet toedienen van een voor de nieren risicovol antibioticum of het switchen naar een nierveilig antibioticum niet mogelijk is, zal echter niet van klinische meerwaarde zijn.

De klinische meerwaarde van de voorspellingen in termen van handelingsopties is dus afhankelijk van de unieke omstandigheden van de patiënt en het moment waarop de voorspelling wordt gegenereerd. Suggesties omvatten het presenteren van onderliggende richtlijnen en wetenschappelijke literatuur bij de modeluitkomsten, om artsen te helpen deze in context te plaatsen en de relevantie voor de betreffende patiënt beter in te schatten. Verder werd al voorgesteld om de presentatie van de uitkomsten te verbeteren, bijvoorbeeld door gebruik te maken van visuele hulpmiddelen zoals de mogelijkheid om op 'informatiebalonnetjes' te kunnen klikken bij behoefte aan meer verdieping en/of andere genuanceerde presentatiemethoden waarbij bijdragen van verschillende potentiële oorzaken van acuut nierfalen worden getoond.

***1.2.6 Retrospectief vs. Prospectief gebruik.*** Ook richtte het gesprek zich op het onderscheid tussen retrospectieve en prospectieve modellen. Het huidige beoogde causale machine learning-model is specifiek ontwikkeld voor achterafdiagnose, waarbij het model de waarschijnlijkheid inschat dat medicatie de oorzaak was van acuut nierfalen. Deelnemers uitten hun zorgen dat in de praktijk er meer behoefte bestaat om een model te hebben dat prospectief een kans op acuut nierfalen door een bepaald medicijn zou kunnen voorspellen. Dan kun je er als zorgverlener nog iets aan doen (bijv. risico beperken door een ander medicijn te kiezen of extra te monitoren). De onderzoekers benadrukten dat het model eerst gericht is op het verzamelen van informatie over reeds ontstane bijwerkingen, omdat er onvoldoende hoogwaardige bijwerkingsdata beschikbaar zijn om prognostische modellen te kunnen maken. Deze retrospectieve aanpak kan daarom gezien worden als

een essentiële eerste stap in de ontwikkeling van een ander model dat wel prospectief risico's op bijwerkingen door medicatie kan voorspellen.

***1.2.7 Het integreren van patiëntendata uit het EPD met data uit andere kennisbronnen***. Er werd opgemerkt dat het toevoegen van meer en diverse vormen van data niet per se leidt tot betere resultaten, maar dat het opnemen van grote hoeveelheden informatie in het model ten koste zou gaan van de specificiteit van een model. Wanneer uitkomsten worden gepresenteerd als gegeneraliseerde of 'gemiddelde' informatie zonder specificiteit, kunnen ze tekortschieten bij het bieden van bruikbare inzichten voor individuele patiënten. Dit zou er onder andere toe kunnen leiden dat zorgverleners modeluitkomsten negeren. Daarnaast werd de aandacht gevestigd op het risico van het versterken van publicatiebias: een vertekening in wetenschappelijke literatuur die ontstaat wanneer voornamelijk onderzoeken met positieve resultaten worden gepubliceerd, terwijl negatieve of niet-conclusieve bevindingen ongepubliceerd blijven.

# Fase 2

## 2.2 Actoren

Dit deel van de workshop richtte zich op het gezamenlijk in kaart brengen van actoren die mogelijk betrokken zijn bij de casus. Hoewel de focus lag op het betrekken van een diverse groep belanghebbenden, heeft niet iedereen direct te maken met het gebruik van routinematig verzamelde zorgdata of een causaal machine learning-model voor achterafdiagnose van acuut nierfalen door medicatie bij patiënten met chronische nierziekte. Dergelijke actoren zijn misschien minder vanzelfsprekend voor de onderzoekers, maar kunnen indirect invloed uitoefenen op de ontwikkeling en implementatie van het model of de gevolgen ervan ondervinden. Door relevante actoren te identificeren, krijgt het LEAPfROG-project waardevolle inzichten in ontbrekende perspectieven. Deze kunnen in latere fasen van het project alsnog worden geraadpleegd.

Tabel 1. *Actoren*

| Categorie | Actoren |
|---|---|
| Patiënten | In het bijzonder: patiënten met chronische nierziekte en hun naasten; (nier-)patiëntorganisaties. |
| Gezondheidszorg | (Huis-)artsen, verplegers, ziekenhuisapothekers, apothekers buiten het ziekenhuis, kwaliteitsfunctionarissen, mantelzorgers. |
| Technologie & Data | Organisaties die routinematig verzamelde zorgdata leveren, gegevensbeheerders, leveranciers van EPD-systemen. |
| Beleid | Amsterdam UMC, om de vertaalslag van wet- en regelgeving naar beleid te maken. |
| Wet en regelgeving | Nationale overheid (bijv. VWS), lokale overheden, Europese Unie. |
| Research | Onderzoekers in het farmacotherapiedomein. |
| Valorisatie | Valorisatie Amsterdam UMC; Innovation Exchange Amsterdam (IXA) (bijv. ondersteuning bij het vermarkten van innovaties). |

| | |
|---|---|
| Financiering | Zorgverzekeraars, onderzoeksfinanciers, (inter)nationale overheden. |
| Toezicht | Bijv. NZa (Nederlandse Zorgautoriteit), functionarissen gegevensbescherming. |
| Commerciële partijen | Farmaceutische industrie, ontwikkelaars en leveranciers van EPD-systemen, ontwikkelaars van Artificial Intelligence (AI)-software, ontwikkelaars van Persoonlijke Gezondheidsomgevingen (PGO). |
| Publieke opinie | Media, belangengroepen. |
| Netwerk/uitwisselingsplek tussen categorieën | Netwerken en knooppunten in het netwerk (bijv. tussen ziekenhuis-informatiesystemen (ZIS), huisarts-informatiesystemen (HIS) en apotheek-informatiesystemen (AIS)). |
| Techniek (niet-menselijke actoren) | Bijv. AI-modellen en EPD-systemen. |

## 2.2 Mogelijke Effecten

Technologieën maken deel uit van een groter geheel waarin verschillende factoren samenkomen. Denk hierbij aan menselijk gedrag, interacties tussen mensen (zoals zorgprofessionals), en omgevingskenmerken zoals regelgeving, normen en fysieke omstandigheden. Dit omvat ook de systemen waarin het LEAPfROG causale machine learning-model wordt ingebed. Al deze elementen beïnvloeden elkaar continu, waardoor het moeilijk is om precies te voorspellen wat er gebeurt wanneer een technologie wordt ingezet. Bovendien kan een effect dat in de ene situatie positief wordt ervaren, in een andere situatie juist onwenselijk zijn. Dit hangt sterk af van de specifieke omstandigheden en de waarden en meningen van de betrokken mensen. Daarom is het belangrijk om goed na te denken over hoe en waar een technologie wordt gebruikt, en ervoor te zorgen dat deze blijft functioneren volgens de wensen en behoeften van de gebruikers. Het onderstaande overzicht van mogelijke positieve en negatieve effecten van het beoogde causale machine learning-model voor achterafdiagnose van medicatiegerelateerd acuut nierfalen bij patiënten met chronische nierziekte, samengesteld tijdens de workshop, dient als startpunt voor verdere verkenning en verdieping binnen het LEAPfROG-project. Het is niet bedoeld als een volledig beeld, maar als een uitnodiging om samen het gesprek voort te zetten.

Tabel 2. *Effecten van het beoogd causaal machine learning model.*

| Positieve effecten | Negatieve effecten |
|---|---|
| Bewustwording rondom bijwerkingen bij zorgverleners én patiënten. | Kwetsbaarheid van de arts: kan mogelijk bedreigend voelen om achteraf geconfronteerd te worden met wat "mis" ging; kwetsbaarheid van de positie van de arts (menselijk oordeel) tegenover een AI-gedreven uitkomst. |
| Bevestiging voor zorgverleners van een vermoeden van een medicatiebijwerking en meer | Zichtbaar voor arts en patiënt dat er iets niet goed is gegaan; kwetsbaarheid in de vorm van liability/aansprakelijkheid. |

| | |
|---|---|
| inzicht in werkingsmechanismen van bijwerkingen. | |
| Biedt patiënten inzicht in de klinische besluitvorming (transparantie zorgproces) over medicatiebijwerkingen. | Stress bij de patiënt door onduidelijkheid over de betekenis en relevantie van het waarschijnlijkheidspercentage dat het model voorspelt. |
| Bevordert communicatie tussen arts en patiënt over medicatiebijwerkingen. | Het model wordt onjuist gebruikt, bijvoorbeeld door het toch prospectief toe te passen, terwijl het specifiek is ontworpen voor retrospectief gebruik. |
| Biedt zorgverleners een leidraad/startpunt in de enorme complexiteit rondom het vaststellen van een medicatiebijwerking. | Schade aan de arts-patiëntrelatie door misverstanden, zoals het idee van schuld ten aanzien van de arts. |
| Meer regie voor patiënten over hun eigen behandeling met medicijnen (autonomie), bijvoorbeeld stoppen met het gebruik van middel X in de toekomst. | *Te grote afhankelijkheid AI-model*; bias ten gunste van data-gedreven uitkomsten, ten koste van klinisch redeneren/besluitvorming van zorgverleners waarin ook niet op data-gebaseerde context wordt meegewogen. |
| Ontwikkeling/aanscherping van ervaring/expertise van zorgverleners bij het detecteren van medicatiebijwerkingen (leereffect model); ondersteunend in klinische besluitvorming. | Te grote afhankelijkheid van het AI-model; bias ten gunste van data-gedreven uitkomsten, ten koste van klinisch redeneren/besluitvorming van zorgverleners, waarin ook niet op data-gebaseerde context wordt meegewogen. |
| Meer inzicht in onderzoeksprioriteiten met betrekking tot medicatieveiligheid. | Te grote afhankelijkheid van het AI-model; verlies van, en gebrek aan ontwikkeling van expertise (*deskilling*) omtrent de diagnose van medicatiebijwerkingen. |
| Een goed werkend model kan een uitstralingseffect hebben en bijvoorbeeld aanleiding geven tot het ontwikkelen van een uitgebreider bijwerkingenprofiel dat verder reikt dan alleen acuut nierfalen. | Het model gebruikt bestaande domeinkennis, zoals wetenschappelijke publicaties over de veiligheid van geneesmiddelen, als input voor de voorspellingen. Het versterken van publicatiebias ligt dan op de loer als er sprake is van meer gepubliceerde studies met positieve effecten van geneesmiddelen dan met negatieve of geen effecten van geneesmiddelen. |
| Biedt mogelijk inzicht in variërende praktijken binnen en tussen zorginstellingen (bijv. patronen in het voorschrijven van bepaalde potentieel nefrotoxische medicatie op de afdeling interne geneeskunde vs. cardiologie). | Meer data leiden niet automatisch tot een betere voorspelling van een causale relatie tussen medicatie en acuut nierfalen; een gebrek aan specificiteit voor een individuele patiënt kan resulteren in een te algemene, gemiddelde voorspelling op populatieniveau, niet geënt op de situatie van een individuele patiënt. |
| Minder bijwerkingen – door het leereffect van achterafdiagnose kan ook een leereffect ontstaan bij zorgverleners, waardoor zij risicovolle situaties beter kunnen inschatten en mogelijk acuut nierfalen door medicatie kunnen voorkomen (kwaliteit van leven). | Voorspellingen die een vorm hebben van ‘gemiddelde’ informatie (gebrek aan specificiteit voor een individuele patiënt) hebben geen klinische meerwaarde. De kans is groot dat artsen deze voorspellingen gaan negeren; kans op signaalmoeheid. |
| Minder bijwerkingen op de nieren – lagere zorgkosten. | Risico op een té intelligent of overtuigend beeld van het model (hoe wordt het gepresenteerd) bij zorgverleners. |

| | |
|---|---|
| Registratielastverlagend, want het model ondersteunt bij het verzamelen van benodigde informatie uit het EPD om medicatiebijwerkingen te signaleren en te registreren; kwalitatief betere registratie van medicatiebijwerkingen in het EPD. | Kan patiënten wantrouwend maken jegens zorgverleners en toekomstig medicatiegebruik (bijvoorbeeld wanneer een middel dat eerder acuut nierfalen veroorzaakte opnieuw wordt voorgeschreven). |
| Beschikking over meer gedegen kennis/informatie over de veiligheid van geneesmiddelen voor de nieren door toepassing van het model op EPD-data. | Verhoging van registratie-/administratielast, omdat het model bijwerkingen detecteert die vervolgens geregistreerd moeten worden (tijdrovend, mogelijk ten koste van werkplezier, en verhoogd risico op incomplete registratie door selectiebias in de registratie van alleen bijwerkingen met een hoge waarschijnlijkheid van een causale relatie). |
| Blinde vlekken in het klinisch redeneren van zorgverleners zichtbaar maken: het model als alternatief of aanvulling op de differentiaaldiagnose, gebaseerd op de parate kennis van zorgverleners. | Onbetrouwbare kennis over de veiligheid van geneesmiddelen voor de nieren bereikt de praktijk als het model niet zorgvuldig wordt ontwikkeld en de EPD-data van onvoldoende kwaliteit is. |
| Duurzaamheid en resources (bijv. eerder stoppen met medicijnen die meer bijwerkingen dan gewenste werking geven). | Reputatieschade voor Amsterdam UMC als het model tot verkeerde medische beslissingen leidt. |
| Kennisontwikkeling in het bredere AI-veld omtrent causale machine-learningmodellen in de zorg. | Risico op vertekening of verschuiving in de manier waarop de voorspellingen van de waarschijnlijkheid op een causaal verband tussen medicatie en acuut nierfalen worden vertaald naar wat de arts te zien krijgt in het EPD. |
| Bewustwording rondom bijwerkingen bij zorgverleners én patiënten. | Duurzaamheid van AI-modellen (resources; klimaatimpact). |
| Bevestiging voor zorgverleners van een vermoeden van een medicatiebijwerking en meer inzicht in werkingsmechanismen van bijwerkingen. | Risico op dataverlies, misbruik en privacy-issues, met name in samenwerking met commerciële partners. |

# Fase 3

## 3.1 Handelingsopties

In de afrondende fase van de workshop werden de uitkomsten van de voorgaande fasen samengebracht en vormden deze de basis voor een verdiepende discussie over mogelijke handelingsopties. Er is een eerste stap gezet om handelingsopties te bedenken die de positieve effecten van het model zoveel mogelijk kunnen waarborgen en de negatieve effecten kunnen beperken. De groep werd ingedeeld in drie subgroepjes, om vanuit elk van de volgende niveaus tot handelingsopties te komen: techniek, omgeving en gebruiker.

Tabel 3. Handelingsopties

| | |
|---|---|
| Techniek: wat kan in het model worden ingebouwd zodat de positieve effecten worden gewaarborgd? | - De presentatie van de voorspellingen door het model moet de complexiteit van causaal redeneren weergeven en zorgverleners tegelijkertijd uitnodigen tot zelfreflectie.<br>- De presentatie van de voorspellingen door het model moet ook begrijpelijk zijn voor de patiënt (inclusief een eventuele disclaimer; hoe de voorspelling te interpreteren; welke informatie is meegenomen en welke niet).<br>- Anticipeer op toekomstig (mis)gebruik van het model (bijvoorbeeld diagnostisch doel tóch prognostisch gebruikt) door heel precies vast te stellen welke EPD-gegevens nodig zijn voor een causale voorspelling en wat het model wel of niet kan. Het is daarbij ook belangrijk om duidelijk te bepalen welke EPD-gegevens níet nodig zijn.<br>- Zorg ervoor dat naast ondersteuning van de zorgverleners bij het registreren van medicatiebijwerkingen, ook de voorspellingen van het model, de modelversie en relevante parameters die de voorspelling beïnvloeden zo veel mogelijk geautomatiseerd worden vastgelegd, om te voorkomen dat belangrijke informatie verloren gaat bij de interpretatie van de geregistreerde bijwerkingen door andere zorgverleners. Denk hierbij bijvoorbeeld aan het kunnen terughalen van een klinische notitie, met daarbij een screenshot of iets dergelijks.<br>- Zorg ervoor dat bij het gebruik van het model de onderliggende domeinkennis (bijvoorbeeld literatuurreferenties) ook inzichtelijk en toegankelijk is voor de eindgebruikers, zodat beoordeeld kan worden of de gegenereerde voorspelling relevant is voor de specifieke patiënt en bijdraagt aan het vaststellen van een causale relatie (bijvoorbeeld in de vorm van een |

| | |
|---|---|
| | geïntegreerde infoknop in het uiteindelijke model).<br>- Bepaal of het model een actieve of een passieve vorm van beslissingsondersteuning moet krijgen (bijvoorbeeld d.m.v. een intrusieve pop-up bij actief of d.m.v. een nazoeklijst op eigen initiatief bij passief).<br><br>- Druk de voorspellingen uit in neutrale termen die het klinisch redeneren van de zorgprofessional ondersteunen. Dit kan bijvoorbeeld worden gefaciliteerd door het gebruik van pop-ups in de uiteindelijke software, zoals: 'Blijf kritisch, stel vraag X, Y, Z.' of door termen als 'verhoogd' of 'verlaagd' te gebruiken in plaats van percentages.<br>- Onderzoek en implementeer verschillende methoden om onzekerheid te meten, zoals het gebruik van statistische significantie en betrouwbaarheidsintervallen, om de interpretatie van de voorspellingen door de causale modellen te verbeteren. Bepaal daarbij tevens welke visuele benadering het meest geschikt en begrijpelijk is voor de eindgebruikers voor de communicatie van onzekerheid.<br>- Bouw alarmeringsniveaus in (pop-ups, kleurcodes) zodat de zorgverlener kan bepalen of interactie met het model nodig is, bijvoorbeeld een groene knop voor optioneel raadplegen en een rode knop bij hoge urgentie.<br>- Stel de gewenste frequentie, het tijdstip en de vorm van de voorspellingen door het model vast.:<br>  ○ Uitkomst over één patiënt of over een groep patiënten. Oftewel: de uitkomsten aangaande één patiënt in al zijn complexiteit of de uitkomsten over een groep patiënten.<br>  ○ Zorgverlener: krijgt hij/zij terugkoppeling van het model één keer per dag, één keer per week, etc.?<br>  ○ Patiënt: sommige patiënten zijn langer opgenomen en dan is het doel mogelijk om een trend vast te stellen.<br>  ○ In welk stadium van het zorgproces interacteer je als zorgverlener met het model? Bij het ontslag? Aan het begin van de dienst? In aanwezigheid van de patiënt?? |

| | |
|---|---|
| Omgeving: wat kunnen de onderzoekers die het model ontwikkelen en de organisatie waarin het model wordt gebruikt doen om de positieve effecten te waarborgen? | - Creëer separate denkstromen met als startpunt dat het model anders redeneert dan een mens, zeker als het model erg complex is en het daardoor moeilijk te doorgronden is hoe het precies tot bepaalde voorspellingen komt.<br>o 1. Zorgverlener staat voor menselijk klinisch redeneren.<br>o 2. Causaal machine learning model staat voor data-gedreven redeneren.<br>o 3. Conclusie zorgverlener en voorspelling door het model komen samen.<br>o 4. Zorgverlener neemt beslissing.<br>o Extra stap (vooral gedurende de onderzoeks- en ontwikkelingsfase van het model): reflectie op de invloed van het model op het klinisch redeneren van de zorgverlener en het daaropvolgende zorgproces, en de beoordeling van causaliteit, uitgevoerd door een groep of ten minste twee zorgverleners (bijvoorbeeld volgens het vierogenprincipe).<br>- Biedt ruimte voor de zorgverlener om te motiveren in het EPD waarom op een bepaalde manier is gehandeld (of het nalaten daarvan) op basis van interactie met het model en de voorspellingen die het model heeft gegenereerd.<br>o Koppel motivaties terug aan onderzoekers, zodat het model waar mogelijk verbeterd kan worden.<br>- Ontwikkel duidelijke richtlijnen voor artsen waarin wordt aangegeven hoe het model gebruikt dient te worden en hoe de voorspellingen door het model moeten worden geïnterpreteerd. Dit voorkomt dat percentages zonder de juiste context bij besluitvorming te serieus worden genomen.<br>- Ontwikkel patiëntvriendelijke informatie over het model met een eenvoudige, duidelijke uitleg van de voorspellingen die het model genereert en de relevantie daarvan voor de patiënt.<br>- Zorg ervoor dat het gebruik van het model ter ondersteuning van causaal redeneren transparant wordt gecommuniceerd aan patiënten en het zorgproces tussen arts en patiënt bevordert, bijvoorbeeld door het |

| | |
|---|---|
| | ontwikkelen van scholing voor zorgverleners en patiënten.<br>- Stem de rol van commerciële partners goed af, zodat onder andere de privacy van patiënten gewaarborgd blijft.<br>- Onderzoekers binnen het LEAPfROG-project moeten kennis hebben van en op de hoogte blijven van ontwikkelingen rondom de Europese AI Act, het AI Impact Assessment-hulpmiddel (AIIA), of bijvoorbeeld een Data Protection Impact Assessment (DPIA) doen. Daarnaast moeten zij samenwerken met onder andere research support, functionarissen privacybescherming en ethici.<br>- Start een campagne of charmeoffensief om de meerwaarde van het causale machine learning-model voor achterafdiagnose over te brengen, zodat het niet belerend overkomt, maar op een positieve manier wordt ontvangen door de beoogde eindgebruikers (met name artsen, apothekers en patiënten). Dit kan bijvoorbeeld door onderzoeksresultaten actief terug te koppelen naar de beoogde eindgebruikers om de relevantie van het model aan te tonen..<br>    o Bijvoorbeeld om de zorgen over onder andere een hogere registratielast omtrent medicatiebijwerkingen en de kwetsbaarheid van de zorgverlener weg te nemen. |
| Gebruiker: hoe kunnen zorgverleners en patiënten die het model gebruiken de positieve effecten waarborgen? | - Zorgverleners moeten scholing volgen over AI in het algemeen (bijv. de werking en risico's bij gebruik).<br>- Zorgverleners moeten scholing volgen over het model: wat het wel en niet kan, en hoe je de resultaten interpreteert. (Link met techniek: bijvoorbeeld een infoknopje met een duidelijke beschrijving: wat zit er in het model? Hoe is het model tot stand gekomen? Hoe betrouwbaar is het model?)<br>- Patiënten moeten kennisnemen van de informatie over het model. Een duidelijke instructiebeschrijving, bedoeld voor de patiënt, kan hierbij helpen (bijvoorbeeld een instructiebeschrijving die de arts aan de patiënt zou kunnen geven).<br>- Artsen en apothekers hebben de plicht om kritische vragen te blijven stellen en geïnformeerd te blijven over wetenschappelijke inzichten uit studies naar |

| | |
|---|---|
| | medicatiebijwerkingen en niet enkel te leunen op de voorspellingen van het model. |

## Reflectie op de Workshop door Onderzoekers van het LEAPfROG-project

De dialoog met de diverse stakeholders tijdens de workshop heeft veel bruikbare aanknopingspunten opgeleverd om de positieve effecten van het beoogde causale machine learning-model te waarborgen. Vooral de zorgen over registratielast, beperkte klinische meerwaarde bij achterafdiagnose en het mogelijke confronterende gevoel dat het model bij eindgebruikers (met name artsen en apothekers) kan oproepen, zijn negatieve effecten. Dit zijn aspecten waaraan wij als onderzoekers extra aandacht zullen besteden. Deze effecten beïnvloeden naar onze mening de beeldvorming van het model op een negatieve manier. Hierdoor bestaat het risico dat eindgebruikers het model niet als zinvolle beslissingsondersteuning zien als het in de toekomst in de praktijk wordt geïmplementeerd.

Verder is het belangrijk dat de beoogde eindgebruikers op een basaal niveau inzicht hebben in hoe het model werkt en hoe de voorspelling van een causaal verband tot stand komt. Dit kan bijvoorbeeld in de vorm van een korte toelichting op de gegenereerde waarschijnlijkheid van een causale relatie. Deze toelichting zou in hetzelfde scherm zichtbaar moeten zijn, zodat het snel kan worden geraadpleegd. Het gebruik van een complex model kan namelijk als een black box worden ervaren, wat het vertrouwen en uiteindelijk de acceptatie bij eindgebruikers kan belemmeren. Het helpt aanzienlijk als zij begrijpen waarom een bepaalde voorspelling van een causaal verband tot stand komt.

Daarnaast moet duidelijk zijn voor de beoogde eindgebruikers welk zorgprobleem het model dient op te lossen. Het gaat hierbij om het vergroten van kennis over acuut nierfalen veroorzaakt door medicatie en niet om het opsporen van 'missers'. Ook is het belangrijk om uit te leggen waarom het noodzakelijk is eerst een achterafdiagnosemodel te ontwikkelen (anders kunnen er geen prognostische modellen worden gemaakt) en hoe het model zorgverleners ondersteunt bij het causale redeneren, gezamenlijke besluitvorming met patiënten en het registreren van bijwerkingen. In de toekomst, wanneer een dergelijk model in de praktijk wordt toegepast, zal het van belang zijn om deze vorm van beslissingsondersteuning continu te evalueren. Dit is nodig om te bepalen hoe het model daadwerkelijk bijdraagt aan de besluitvorming. Hierdoor kan het model iteratief worden verbeterd, zodat wantrouwen en een mogelijk confronterend gevoel bij eindgebruikers afnemen en er juist meer begrip en acceptatie ontstaan.

Vroegtijdige initiatieven om samenwerking met eindgebruikers te versterken, zoals deze fysieke begeleidingsethiekworkshop, dragen naar onze mening sterk bij aan het succes van het beoogde causale machine learning-model. Dergelijke sessies helpen namelijk om de autonomie van eindgebruikers in het proces te behouden. Dit voorkomt dat zij nieuwe modellen als bedreigend ervaren en bevordert juist een gevoel van ondersteuning. Het begrijpen van elkaars wereld en werkmethoden blijft echter een complexe uitdaging. De meerwaarde van dit soort sessies ligt daarom ook in het faciliteren van wederzijdse dialoog over uiteenlopende perspectieven. Deze uitwisseling is cruciaal, omdat verschillende stakeholders vaak vanuit hun eigen dagelijkse realiteit en verantwoordelijkheden naar dezelfde situatie kijken. Dit kan leiden tot uiteenlopende interpretaties.

De workshop heeft ons geholpen om de eerste contouren te schetsen van een gezamenlijke visie en mogelijke oplossingsrichtingen. Hiermee werken we toe naar een waardevolle inzet van AI voor de diagnose van bijwerkingen.

## BIJLAGE 1. DEELNEMERS

| | |
|---|---|
| Joanna Klopotowska | universitair docent, principal investigator en projectleider LEAPfROG, afd. Klinische Informatiekunde, Amsterdam UMC |
| Menno Maris | PhD-kandidaat medische ethiek, Amsterdam UMC |
| Mariëtte van den Hoven | hoogleraar medische ethiek, Amsterdam UMC |
| Marieke Bak | medisch ethicus en universitair docent, Amsterdam UMC |
| Joris Lieverse | PhD-kandidaat LEAPfROG, afd. Klinische Informatiekunde, Amsterdam UMC |
| Giovanni Cina | universitair docent, afd. Klinische Informatiekunde Amsterdam UMC & AI Specialist, Pacmed |
| Renée de Wildt | patiëntvertegenwoordiger, Nierpatiënten Vereniging Nederland (NVN) |
| Anat Fuldauer | functionaris gegevensbescherming, Sigra |
| David de Koning | Research data management, Amsterdam UMC |
| Marjolijne Markvoort | praktijkhoudend huisarts |
| Oscar van der Meer | AI engineer, Amsterdam UMC |
| Juul Aarts | projectapotheker, Amsterdam UMC |
| Eline Tuinen | projectapotheker, Amsterdam UMC |
