# Supplementary material for: The Ethics of Leveraging Routinely Collected Patient Data for AI Development: Mixed Methods Study
Source: J Med Internet Res. 2026 Mar 2;28:e79863. doi: 10.2196/79863 (PMC12954709; doi:10.2196/79863)
Supplement: Multimedia Appendix 4 [file jmir-v28-e79863-s004.pdf]

Multimedia appendix 4. Summary table of literature on ethics of routinely collected patient data for AI

- Studies are listed alphabetically by first author.

Author(s), year	Country (corresponding author)	Article type		Study aim	Data type(s) discussed	AI-related focus and key themes
Alami et al. (2020)	Canada	Commentary / viewpoint	General Healthcare	To provide a holistic analysis of AI's real-world value using the health technology assessment core model, supporting decision-makers in guiding responsible deployment and fostering innovation.	Electronic health records (EHR) data, including exploration of integration of medical records, audio, videos, and socioeconomic data, along with enhancing data linkage across hospitals and healthcare providers, including public-private partnerships.	Explores how AI can transform healthcare systems at large, focusing on policy, infrastructure, and care models. It uses the Health Technology Assessment framework to understand system-wide implications of AI integration.
Anom (2020)	United States of America	Commentary (conceptual analysis)	General Medicine / Bioethics	To analyze ethical challenges posed by big data and AI in medicine, including risks to the four principles of biomedical ethics, and to propose mitigation strategies and policy responses.	Health data including EHR data, genetic data, wearable device data, clinical and research data, and social determinants of health data.	Examines how big data and AI challenge core bioethical principles (autonomy, beneficence, nonmaleficence, justice), addressing privacy, consent, bias, and accountability while calling for stronger governance and policy safeguards.

Atkinson & Atkinson (2023)	United States of America	Commentary/ perspective	Healthcare / Biomedical data science / Health equity	To explore the potential of ML and AI to enhance healthcare, while identifying and addressing challenges, including biases and disparities in data that could impact equitable implementation.	Genomic data, EHR data, phenotypic data, administrative and billing data, metadata from surveys and medical records.	Discusses ethical and equity risks in medical ML, especially data bias and lack of diversity in large datasets. Advocates for representative data and cautious implementation of ML tools in healthcare.
Baric-Parker et al. (2020)	United States of America	Essay	General Healthcare (Ethics)	To analyze ethical and social challenges in EHR data-sharing collaborations for AI development, evaluating them against the guiding principles of the ERDs and the RCAIE, and proposing revisions to address these concerns more directly.	EHR data	Examines ethical challenges of sharing patient health data, with particular attention to Catholic healthcare systems and Catholic ethical frameworks, for AI development. Highlights the importance of transparency and trust in partnerships between hospitals and tech firms.
Bednorz et al. (2023)	Sweden	Narrative review	Geriatrics	To, by drawing on expert opinions, evaluate the benefits and challenges of electronic medical records (EMRs) in gerontology, focusing on their potential to enhance care and research through AI and machine learning, while addressing associated ethical and practical considerations.	EMR data, including structured and unstructured clinical and sociodemographic data used in geriatric research and care.	Reviews how AI tools can help improve care for older adults by analyzing electronic medical records, supporting diagnosis and management of conditions like dementia, frailty, and medication risks.

Bozkurt et al. (2020)	United States of America	Review article (systematic review)	Clinical Informatics / Health Equity	To examine the reporting of demographic data and the representativeness of ML models developed using EHR data, particularly those designed for clinical decision-making.	EHR data used for ML model development and clinical decision support.	Evaluates demographic reporting and representativeness in EHR-based ML studies, highlighting risks of bias, limited reproducibility, and poor external validation. Calls for improved reporting standards and equitable data inclusion.
Breen et al. (2019)	United States of America	Narrative review	Public Health / Health Disparities	Examines how big data and data science approaches can accelerate translational research to reduce health disparities. Emphasizes integrating diverse data sources, iterative translational cycles, and attention to ethical and algorithmic bias.	EHRs and the integration of different data sources, e.g., Geospatial data; Cohort data.	Investigates how AI and large datasets can help reduce health disparities by better understanding social and environmental influences on health, especially through local, community-based interventions.
Chekroud et al. (2021)	United States of America	Special article (review article)	Psychiatry / Mental Health	Reviews the use of machine learning to predict treatment outcomes in psychiatry across medications, psychotherapies, and digital interventions. Discusses data sources, validation standards, implementation challenges, and ethical considerations.	Primarily discusses EHRs while also addressing broader clinical data. Genetic data and neuroimaging are highlighted as well.	Machine learning to predict how individuals with depression might respond to different psychological therapies, aiming to personalize mental health care and improve treatment outcomes.

Cohen et al. (2014)	United States of America	Commentary / policy analysis	General Clinical Medicine / Health Systems	To explore the ethical, legal, and policy challenges of using predictive analytics in healthcare and suggests strategies to address them during model development and implementation.	EHRs most predominantly. Clinical data, social data, genomic data, and administrative data are also highlighted.	Analyzes legal and ethical concerns related to using predictive analytics in high-stakes clinical decisions (e.g., ICU admissions), emphasizing the need for governance and responsible oversight of real-time predictive models.
Darcel et al. (2023)	Canada	Original research (qualitative study)	Primary Care	To identify the barriers perceived by patients, providers, and health leaders to implementing AI in Canadian primary care and to propose strategies to overcome them.	Primarily discusses longitudinal data derived from EMRs.	Identifies stakeholder perspectives on AI use in Canadian primary care, including early applications such as risk prediction and decision support, and highlights barriers related to trust, regulation, system readiness, and equity.
Fischer et al. (2016)	Germany	Commentary / debate article	Medical Ethics / Systems Medicine	To explore the ethical and epistemological challenges of systems medicine in clinical practice, focusing on the complexities of data-driven tools and predictive algorithms and the integration of diverse data sources like big data and EHR data, to responsibly anticipate and manage potential issues.	Genomic data, EHRs, imaging data, patient collected data and their role in enriching EHRs.	Critically examines ethical and epistemological challenges of systems medicine, including the use of data-driven algorithms, clinical scoring systems, and secondary findings. Raises concerns about patient autonomy, trust, and the use of “black box” predictive models in clinical decision making.

Ford et al. (2020)	United Kingdom	Original research (qualitative study)	Medical Informatics / Ethics	To explore public opinions on the ethical implications and feasibility of sharing unstructured free-text data from electronic medical records for health research using a citizens' jury to gather input on its benefits, risks, and safeguards.	Free-text health data found in EMRs, including unstructured clinical information such as narrative notes, letters, reports, and comments.	Investigates public attitudes toward sharing free-text clinical notes for health research, emphasizing the need for public trust, transparent data use, and improved anonymization of sensitive information.
Gianfrancesco et al. (2018)	United States of America	Commentary / special communicatio n	General Healthcare / Health Equity	To examine the potential biases in machine learning algorithms used in clinical decision support tools that rely on EHR data.	EHR data, including clinical records, patient-reported data, and linked data from communicating sources (e.g., sensors).	Highlights how ML models trained on EHR data can perpetuate or amplify existing health disparities, emphasizing fairness, representativeness, and transparency.
Ho & Caals (2021)	Hong Kong	Commentary / perspective	Nephrology / Ethics & Governance of Digital Health	To address the fragmented development of digitalization in nephrology by proposing an integrative ethics and governance framework centered on healthcare providers and AI/ML applications.	EHR data, wearable and medical device data, physiological data, and registry/public health data.	Discusses the fragmented digitalization of nephrology and the early-stage development of AI/ML tools in kidney care. Proposes a dedicated, provider-centred ethics and governance framework to guide responsible development, validation, and implementation of AI in nephrology.
Kamradt et al. (2022)	Germany	Original research article (qualitative)	General Practice / Health Services	To explore physicians' perspectives on providing and utilizing real-world care data for the development of artificial intelligence (AI)-based technologies in healthcare within Germany.	Deidentified real-world care data derived from routine patient care in primary care and hospital settings.	Explores physicians' perspectives on donating real-world care data for AI development, highlighting support for patient-centered benefits alongside concerns about transparency, governance, and third-party involvement.

Khalid et al. (2023)	Pakistan	Review article	Medical Informatics / Privacy & Security	To review privacy risks, attacks, and state-of-the-art privacy-preserving techniques for AI in healthcare, and to discuss associated challenges and future directions.	Healthcare data including EHRs, genomic data, medical imaging, and wearable device data.	Reviews privacy-preserving AI approaches (e.g., federated learning, differential privacy, cryptographic methods), outlines privacy attacks and security risks, and discusses trade-offs between privacy, utility, and innovation.
Knevel & Liao (2023)	Netherlands	Review article	Rheumatology	To explore the opportunities and challenges of using real-world data (RWD), particularly EHR data, in advancing clinical research in rheumatology through the application of AI and ML methods.	Real-world data, primarily EHR data including structured and unstructured clinical information.	Reviews how AI and ML can leverage EHR-based real-world data for phenotyping, prediction modeling, and clinical research in rheumatology, while discussing data quality, bias, and implementation challenges.
Landau et al. (2022)	United States of America	Commentary / perspective	Pediatrics / Child Protection	To examine ethical challenges and propose recommendations for developing ML-based models to identify child abuse and neglect using pediatric EHR data.	Pediatric emergency department EHR data	Discusses ethical challenges in using ML to detect child abuse and neglect from EHR data, emphasizing fairness, explainability, bias mitigation, and minimizing harm from false positives.

Liaw et al. (2020)	Australia	Review article (expert consensus article)	Primary Care / Medical Informatics	To provide practical recommendations for the ethical curation and use of longitudinal health data derived from Electronic Medical Records (EMRs) and Artificial Intelligence (AI) in primary care.	Routinely collected health data from primary care settings, primarily from EHRs, but discusses linkage with external datasets (e.g., disease registries; public health data; biobanks)	Provides practical recommendations for ethical data curation and governance across the health data lifecycle in primary care, including AI-related use. Emphasizes consent, trust, data quality, and responsible secondary use in integrated care systems.
Liu and Panagiotakos (2022)	United States of America	Review article	Clinical research methodology / Data science	To provide a primer on real-world data, including its types, analytical approaches, challenges, and opportunities for evidence-based decision making.	Real-world data sources including EHRs, registry data, claims and billing data, patient-reported outcomes, wearable device data, and social media data.	Reviews the use of real-world data for evidence generation, discussing methodological approaches, data quality challenges, causal inference, and the responsible use of AI/ML in analysis.
Lovis (2019)	Switzerland	Commentary / viewpoint	Medical Informatics / Policy	To examine the transformative potential of AI and big data in medicine while highlighting regulatory, ethical, and societal dependencies required for responsible adoption.	Big data sources including clinical data, EHR data, medical imaging, and genomic data.	Discusses how AI and big data can advance medicine while emphasizing the need to address legal, ethical, regulatory, and data quality challenges. Highlights trust, transparency, and alignment with societal values as prerequisites for adoption.

Müller (2022)	Germany	Commentary / debate article	Medical Ethics / Public Health	To critically evaluate three arguments in favor of a civic duty to share electronic health records (EHRs) for medical AI development: the "rule to rescue," the "low risks, high benefits," and the "property rights" arguments.	EHR data, including identifiable and re-identifiable patient data.	Argues against a general moral duty to share EHRs for medical AI and instead proposes a context-sensitive civic responsibility model grounded in public deliberation.
Paulus and Kent (2020)	United States of America	Commentary / perspective	Clinical prediction / Health disparities	To explore how clinical prediction models may unintentionally worsen health disparities, distinguishing between algorithmic fairness and bias, especially in healthcare resource allocation. They propose a framework to address these issues.	Clinical and administrative health data (e.g., EHR data, outcome data, and demographic/socioeconomic variables).	Analyzes how clinical prediction models may introduce bias or unfairness that worsens health disparities. Proposes a framework distinguishing algorithmic bias from fairness and outlining strategies to evaluate and mitigate both.
Rubinger et al. (2023)	Canada	Review article (narrative review)	Clinical Research / Orthopedics	To review applications of AI and ML in healthcare research and clinical care, with attention to data quality, interpretability, and ethical considerations.	EMR data, imaging and radiology data, registry data, and clinical/demographic data.	Reviews how AI and ML support clinical decision-making and research, particularly in orthopedics, while emphasizing interpretability, transparency, data quality, and ethical development.

Vollmer et al. (2020)	United Kingdom	Commentary / research methods and reporting article	Clinical AI / Health informatics methodology	To propose a framework of 20 critical questions (TREE: transparency, reproducibility, ethics, effectiveness) to guide the design, evaluation, and reporting of ML/AI research for patient benefit.	Health data sources including EHRs, imaging data, genomic data, registries, and other routinely collected health data.	Outlines a structured set of questions (TREE) to improve the quality and reliability of AI research in healthcare, aiming to promote trustworthy and effective use of machine learning in clinical settings.

References

Alami H, Lehoux P, Auclair Y, et al. Artificial intelligence and health technology assessment: anticipating a new level of complexity. J Med Internet Res. 2020;22(7):e17707. doi:10.2196/17707

Anom BY. Ethics of Big Data and artificial intelligence in medicine. Ethics Med Public Health. 2020;15:100568. doi:10.1016/j.jemep.2020.100568

Atkinson JG, Atkinson EG. Machine learning and health care: potential benefits and issues. J Ambul Care Manage. 2023;46(2):114–120. doi:10.1097/JAC.0000000000000453

Baric-Parker J, Anderson EE. Patient data-sharing for AI: ethical challenges, Catholic solutions. Linacre Q. 2020;87(4):471–481. doi:10.1177/0024363920922690

Bednorz A, Mak JKL, Jylhävä J, Religa D. Use of electronic medical records in gerontology: benefits, considerations and a promising future. Clin Interv Aging. 2023;18:2171–2183. doi:10.2147/CIA.S400887

Bozkurt S, Cahan EM, Seneviratne MG, et al. Reporting of demographic data and representativeness in machine learning models using electronic health records. J Am Med Inform Assoc. 2020;27(12):1878–1884. doi:10.1093/jamia/ocaa164

Breen N, Berrigan D, Jackson JS, et al. Translational health disparities research in a data-rich world. Health Equity. 2019;3(1):588–600. doi:10.1089/heq.2019.0042

Chekroud AM, Bondar J, Delgadillo J, et al. The promise of machine learning in predicting treatment outcomes in psychiatry. World Psychiatry. 2021;20(2):154–170. doi:10.1002/wps.20882

Cohen IG, Amarasingham R, Shah A, Xie B, Lo B. The legal and ethical concerns that arise from using complex predictive analytics in health care. Health Aff. 2014;33(7):1139–1147. doi:10.1377/hlthaff.2014.0048

Darcel K, Upshaw T, Craig-Neil A, et al. Implementing artificial intelligence in Canadian primary care: barriers and strategies identified through a national deliberative dialogue. PLoS One. 2023;18(2):e0281733. doi:10.1371/journal.pone.0281733

Fischer T, Brothers KB, Erdmann P, Langanke M. Clinical decision-making and secondary findings in systems medicine. BMC Med Ethics. 2016;17:32. doi:10.1186/s12910-016-0113-5

Ford E, Oswald M, Hassan L, et al. Should free-text data in electronic medical records be shared for research? A citizens' jury study in the UK. J Med Ethics. 2020;46(6):367–377. doi:10.1136/medethics-2019-105472

Gianfrancesco MA, Tamang S, Yazdany J, Schmajuk G. Potential biases in machine learning algorithms using electronic health record data. JAMA Intern Med. 2018;178(11):1544–1547. doi:10.1001/jamainternmed.2018.3763

Ho CL, Caals K. A call for an ethics and governance action plan to harness the power of artificial intelligence and digitalization in nephrology. Semin Nephrol. 2021. doi:10.1016/j.semnephrol.2021.05.009

Kamradt M, Poß-Doering R, Szecsenyi J. Exploring physician perspectives on using real-world care data for the development of AI-based technologies in health care: qualitative study. JMIR Form Res. 2022;6(5):e35367. doi:10.2196/35367

Khalid N, Qayyum A, Bilal M, Al-Fuqaha A, Qadir J. Privacy-preserving artificial intelligence in healthcare: techniques and applications. Comput Biol Med. 2023;158:106848. doi:10.1016/j.compbiomed.2023.106848

Knevel R, Liao KP. From real-world electronic health record data to real-world results using artificial intelligence. Ann Rheum Dis. 2023;82(3):306–311. doi:10.1136/ard-2022-222626

Landau AY, Ferrarello S, Blanchard A, et al. Developing machine learning-based models to help identify child abuse and neglect: key ethical challenges and recommended solutions. J Am Med Inform Assoc. 2022;29(3):576–580. doi:10.1093/jamia/ocab286

Liaw ST, Liyanage H, Kuziemsky C, et al. Ethical use of electronic health record data and artificial intelligence. Yearb Med Inform. 2020;29(1):51–57. doi:10.1055/s-0040-1701980

Liu F, Panagiotakos D. Real-world data: a brief review of the methods, applications, challenges and opportunities. BMC Med Res Methodol. 2022;22(1):287. doi:10.1186/s12874-022-01768-6

Lovis C. Unlocking the power of artificial intelligence and big data in medicine. J Med Internet Res. 2019;21(11):e16607. doi:10.2196/16607

Müller S. Is there a civic duty to support medical AI development by sharing electronic health records? BMC Med Ethics. 2022;23(1):134. doi:10.1186/s12910-022-00871-z

Paulus JK, Kent DM. Predictably unequal: understanding and addressing concerns that algorithmic clinical prediction may increase health disparities. NPJ Digit Med. 2020;3:99. doi:10.1038/s41746-020-0304-9

Rubinger L, Gazendam A, Ekhtiari S, Bhandari M. Machine learning and artificial intelligence in research and healthcare. Injury. 2023;54(Suppl 3):S69–S73. doi:10.1016/j.injury.2022.01.046

Vollmer S, Mateen BA, Bohner G, et al. Machine learning and artificial intelligence research for patient benefit: 20 critical questions. BMJ. 2020;368:l6927. doi:10.1136/bmj.l6927

Abbreviations: AI, artificial intelligence; EHR, electronic health record; EMR, electronic medical record; RWD, real-world data; ML, machine learning; NLP, natural language processing; ICU, intensive care unit; PROs, patient-reported outcomes; TREE, transparency, reproducibility, ethics, and effectiveness.
